# Identification of the Mechanisms Causing Reversion to Virulence in an Attenuated SARS-CoV for the Design of a Genetically Stable Vaccine

**DOI:** 10.1371/journal.ppat.1005215

**Published:** 2015-10-29

**Authors:** Jose M. Jimenez-Guardeño, Jose A. Regla-Nava, Jose L. Nieto-Torres, Marta L. DeDiego, Carlos Castaño-Rodriguez, Raul Fernandez-Delgado, Stanley Perlman, Luis Enjuanes

**Affiliations:** 1 Department of Molecular and Cell Biology, Centro Nacional de Biotecnología (CNB-CSIC), Campus Universidad Autónoma de Madrid, Madrid, Spain; 2 Department of Microbiology, University of Iowa, Iowa City, Iowa, United States of America; University of Bonn, GERMANY

## Abstract

A SARS-CoV lacking the full-length E gene (SARS-CoV-∆E) was attenuated and an effective vaccine. Here, we show that this mutant virus regained fitness after serial passages in cell culture or *in vivo*, resulting in the partial duplication of the membrane gene or in the insertion of a new sequence in gene 8a, respectively. The chimeric proteins generated in cell culture increased virus fitness *in vitro* but remained attenuated in mice. In contrast, during SARS-CoV-∆E passage in mice, the virus incorporated a mutated variant of 8a protein, resulting in reversion to a virulent phenotype. When the full-length E protein was deleted or its PDZ-binding motif (PBM) was mutated, the revertant viruses either incorporated a novel chimeric protein with a PBM or restored the sequence of the PBM on the E protein, respectively. Similarly, after passage in mice, SARS-CoV-∆E protein 8a mutated, to now encode a PBM, and also regained virulence. These data indicated that the virus requires a PBM on a transmembrane protein to compensate for removal of this motif from the E protein. To increase the genetic stability of the vaccine candidate, we introduced small attenuating deletions in E gene that did not affect the endogenous PBM, preventing the incorporation of novel chimeric proteins in the virus genome. In addition, to increase vaccine biosafety, we introduced additional attenuating mutations into the nsp1 protein. Deletions in the carboxy-terminal region of nsp1 protein led to higher host interferon responses and virus attenuation. Recombinant viruses including attenuating mutations in E and nsp1 genes maintained their attenuation after passage *in vitro* and *in vivo*. Further, these viruses fully protected mice against challenge with the lethal parental virus, and are therefore safe and stable vaccine candidates for protection against SARS-CoV.

## Author Summary

Zoonotic coronaviruses, including SARS-CoV, Middle East respiratory syndrome (MERS-CoV), porcine epidemic diarrhea virus (PEDV) and swine delta coronavirus (SDCoV) have recently emerged causing high morbidity and mortality in human or piglets. No fully protective therapy is still available for these CoVs. Therefore, the development of efficient vaccines is a high priority. Live attenuated vaccines are considered most effective compared to other types of vaccines, as they induce a long-lived, balanced immune response. However, safety is the main concern of this type of vaccines because attenuated viruses can eventually revert to a virulent phenotype. Therefore, an essential feature of any live attenuated vaccine candidate is its stability. In addition, introduction of several safety guards is advisable to increase vaccine safety. In this manuscript, we analyzed the mechanisms by which an attenuated SARS-CoV reverted to a virulent phenotype and describe the introduction of attenuating deletions that maintained virus stability. The virus, engineered with two safety guards, provided full protection against challenge with a lethal SARS-CoV. Understanding the molecular mechanisms leading to pathogenicity and the *in vivo* evaluation of vaccine genetic stability contributed to a rational design of a promising SARS-CoV vaccine.

## Introduction

Coronaviruses (CoVs) are pathogens responsible for a wide range of existing and emerging diseases in humans and other animals [1]. A novel coronavirus causing the severe acute respiratory syndrome (SARS-CoV) was identified in Southeast China in 2002. SARS-CoV rapidly spread worldwide to more than 30 countries within six months, infecting 8000 people and leading to death in approximately 10% of the cases [2, 3]. While SARS-CoV has not reappeared in humans, CoVs including those similar to SARS-CoV, are widely disseminated in bats circulating all over the world, making future SARS-CoV outbreaks possible [4–7]. Furthermore, in September 2012, a novel coronavirus infecting humans, the Middle East respiratory syndrome coronavirus (MERS-CoV), was identified in two patients with severe respiratory disease in Saudi Arabia [8, 9], again indicating that emergence of other highly pathogenic CoVs is likely. Thus, development of efficacious and safe vaccines and anti-virus therapies for these pathogens is essential.

SARS-CoV is an enveloped virus with a positive sense RNA genome of 29.7 kb that belongs to the *Coronavirinae* subfamily, genus *β* [2]. The virion envelope contains embedded three structural proteins, spike (S), envelope (E), and membrane (M) and several group specific proteins: 3a, 3b, 6, 7a, and 7b [10–12]. The S protein, which mediates virus entry into host cells, the 3a protein and the M proteins, induce neutralizing antibodies, with those specific for S protein being most protective [13–16]. The SARS-CoV S and N proteins trigger T cell responses [17], which are also important for protection and enhance the kinetics of virus clearance [18, 19].

SARS-CoV E protein is a small integral membrane protein of 76 amino acids that contains a short hydrophilic amino-terminus followed by a hydrophobic transmembrane domain and a hydrophilic carboxy-terminus [20]. E protein oligomerizes to form an ion-conductive pore in membranes [21–23], and contains a PDZ-binding motif (PBM) formed by its last four carboxy-terminal amino acids [24, 25]. PDZ domains are protein-protein recognition sequences, consisting of 80 to 90 amino acids that bind to peptide sequences (PBMs) [26–28]. These protein-protein interactions modulate cellular pathways important for viral replication, dissemination in the host and pathogenesis [29]. We previously demonstrated that a SARS-CoV lacking the E gene (SARS-CoV-∆E) was attenuated in different animal models [30–34], indicating that SARS-CoV E protein is a virulence factor. SARS-CoV lacking the E protein fully protected both young and elderly BALB/c mice against challenge with virulent mouse-adapted SARS-CoV [32]; therefore, rSARS-CoV-∆E is a promising vaccine candidate. Live attenuated vaccines are considered highly effective because of their ability to replicate within host cells, resulting in high levels of antigenic stimulation, and robust long-term immunological memory [35, 36].

However, a major safety concern with live attenuated vaccines is the possibility of reversion to a pathogenic form. CoVs are prone to RNA recombination and mutation in tissue culture and during animal infection [37], so it is crucial that rSARS-CoV-∆E be thoroughly studied after serial passage. In this study, we show that passage of viruses lacking all or part of the E protein in Vero E6 cells and mouse DBT-mACE2 cells [38] led to the incorporation of compensatory insertions.

Interestingly, passage of SARS-CoVs lacking the E protein PBM led to regeneration of viral proteins containing PBMs, either by incorporation of novel chimeric proteins or by the insertion of new PBMs into existing proteins, such as the 8a protein or the E protein if E was only partially deleted. Strikingly, these modifications were not observed after passage of SARS-CoVs with mutated E protein that retained the PBM. In fact, we have shown that if instead of deleting the full-length E protein, only small deletions of 8–12 amino acids were introduced into the carboxy-terminus of E protein with retention of the PBM, the SARS-CoVs generated were attenuated and genetically stable both in cell culture or in mice. While this partial E protein deletion resulted in virus stability, we also augmented vaccine safety by introducing mutations into the SARS-CoV nsp1 protein. The nsp1 protein of CoVs suppresses host gene expression by inducing host mRNA degradation and inhibiting protein translation [39–44], and is an IFN antagonist [45–47]. Nsp1 deletions resulted in attenuated murine coronaviruses that fully protected against the challenge with parental virus [48, 49]. In this manuscript we show that small deletions within SARS-CoV nsp1 protein resulted in virus attenuation, associated with reduction of inflammation and higher levels of IFN-β and interferon-stimulated genes (ISGs). Vaccination with mutated nsp1 variants protected against challenge with the virulent mouse-adapted SARS-CoV (rSARS-CoV) virus. To generate safer vaccine candidates, viruses incorporating deletions in both the nsp1 and E proteins were constructed. These double mutants were protective against virulent virus challenge, and were genetically stable.

## Results

### Stability of recombinant SARS-CoVs lacking the E protein in cell culture

**Fig 1:**
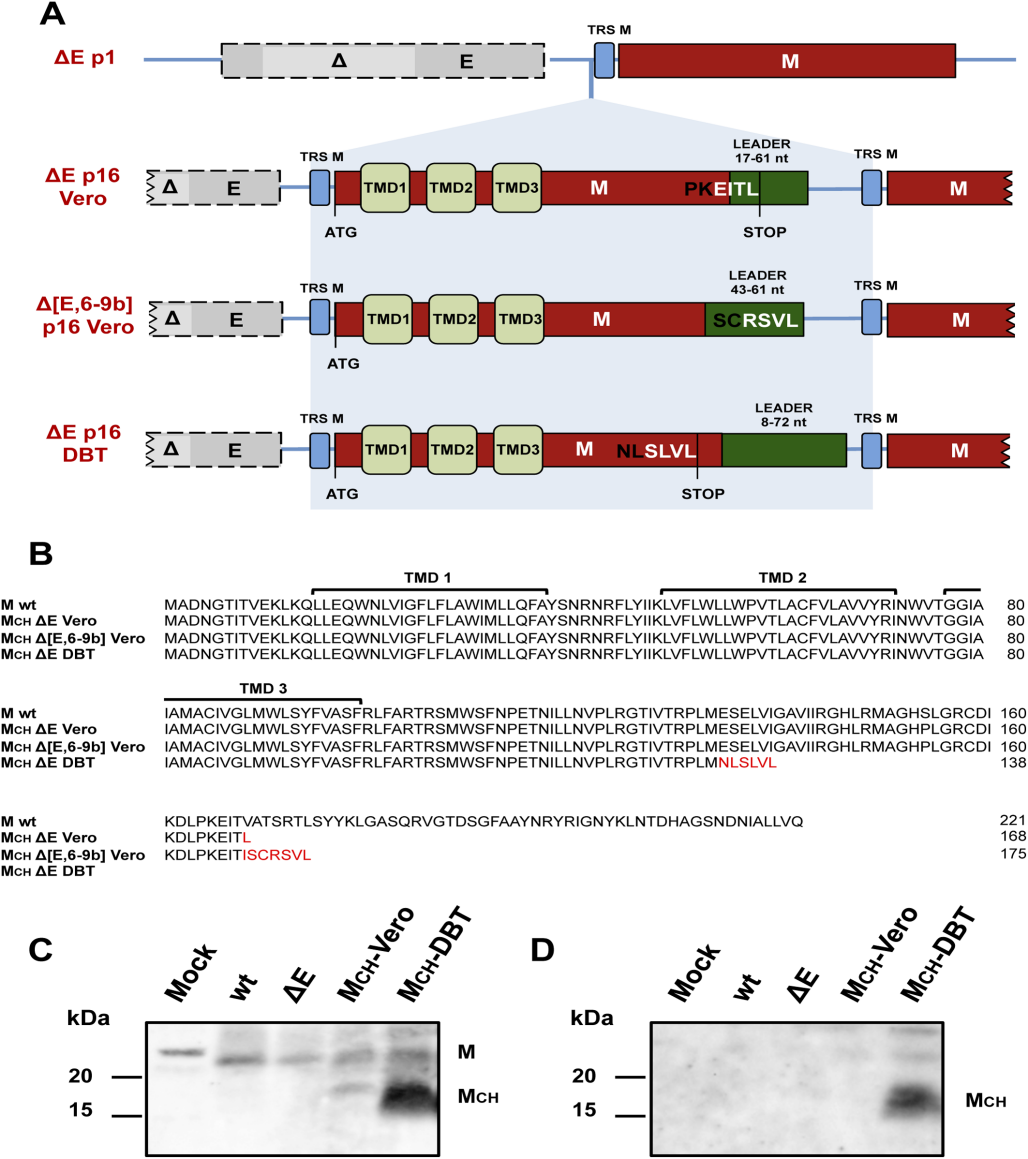
Generation of chimeric membrane genes after 16 serial passages of SARS-CoV lacking E protein in cell culture. (A) Representation of membrane chimeric genes generated after passage of SARS-CoV-∆E (∆E) and SARS-CoV∆[E,6-9b] (∆[E,6-9b] 16 times in Vero E6 cells and ∆E virus in DBT-mACE2 cells. Top, ∆E p1 represents the genomic sequence of viruses lacking E protein at passage 1. Grey boxes indicate E gene with a partial deletion highlighted in light grey (∆). Transcription-regulating sequences (TRSs) of the different genes are shown in blue boxes and the membrane gene (M) is shown in red. Chimeric membrane genes generated after 16 serial passages (p16) are formed by a partial duplication of membrane gene fused to part of SARS-CoV leader sequence (green boxes). TMD1, TMD2 and TMD3 represent the three different transmembrane domains contained within the first part of membrane gene. ATG and STOP represent the start and stop codon of potential proteins, respectively. EITL, RSVL and SLVL represent the last four amino acids of chimeric proteins that form PDZ-binding motifs. (B) Amino acid sequences corresponding to chimeric proteins generated after SARS-CoV-∆E passage in cell culture. New amino acids are shown in red. Presence of native membrane and chimeric membrane proteins was analyzed by Western blot at 24 hpi using two polyclonal antibodies generated to recognize either all membrane proteins, chimeric or not (C) or a unique sequence in the chimeric protein generated after SARS-CoV-∆E passage in DBT-mACE2 cells (D).

To determine the stability of SARS-CoV-∆E, or of virus containing deletions of the E protein and several group specific genes including 6, 7a, 7b, 8a, 8b and 9b (SARS-CoV-∆[E,6-9b]), we infected Vero E6 and DBT-mACE2 cells with rSARS-CoV, rSARS-CoV-∆E or rSARS-CoV-∆[E,6-9b]. Supernatants were serially passaged 16 times and the distal third of the genome, from the S gene to the 3´ end (around 8 kb), was sequenced using specific primers (S1 Table). In all cases, an insertion consisting of a partially duplicated M gene fused to the SARS-CoV leader RNA sequence, a 5´ sequence common to coronavirus mRNAs [50–52] was detected upstream of the native M protein (Fig 1A). In contrast, no chimeric proteins were detected after serial passage of the parental virus. All MCH genes encoded the amino terminus and the three transmembrane domains of M and also different PDZ-binding motifs at the carboxy-terminus of the protein (Fig 1B). Genomic evolution occurred rapidly, as the chimeric genes were already detected within 5 passages in both cells lines, Vero E6 and DBT-mACE2.

The expression of viral sgmRNAs corresponding to the chimeric genes was characterized by RT-PCR (S1A Fig). We used RNA harvested from infected cells after serial passage, plaque purification and amplification along with specific primers (S2 Table). PCR products corresponding to specific MCH sgmRNAs were identified in MCH-Vero and MCH-DBT-infected cells (S1B Fig). Expression of the chimeric proteins encoded by these sgmRNAs was confirmed using an antibody specific for all M and MCH proteins and a second one that recognized the MCH-DBT protein. Vero E6 cells were mock infected or infected with different recombinant viruses (rSARS-CoV, rSARS-CoV-∆E, rSARS-CoV-∆E-MCH-Vero and rSARS-CoV-∆E-MCH-DBT) at a moi of 0.3. The expression of native M and MCH was confirmed at 24 hpi by Western blot analysis (Fig 1C and 1D). These results indicated that, after serial passages of SARS-CoVs lacking the E protein in cell culture, a similar type of chimeric membrane protein was generated in three independent experimental settings.

### Growth kinetics of SARS-CoV-∆E containing MCH genes in cell culture

**Fig 2:**
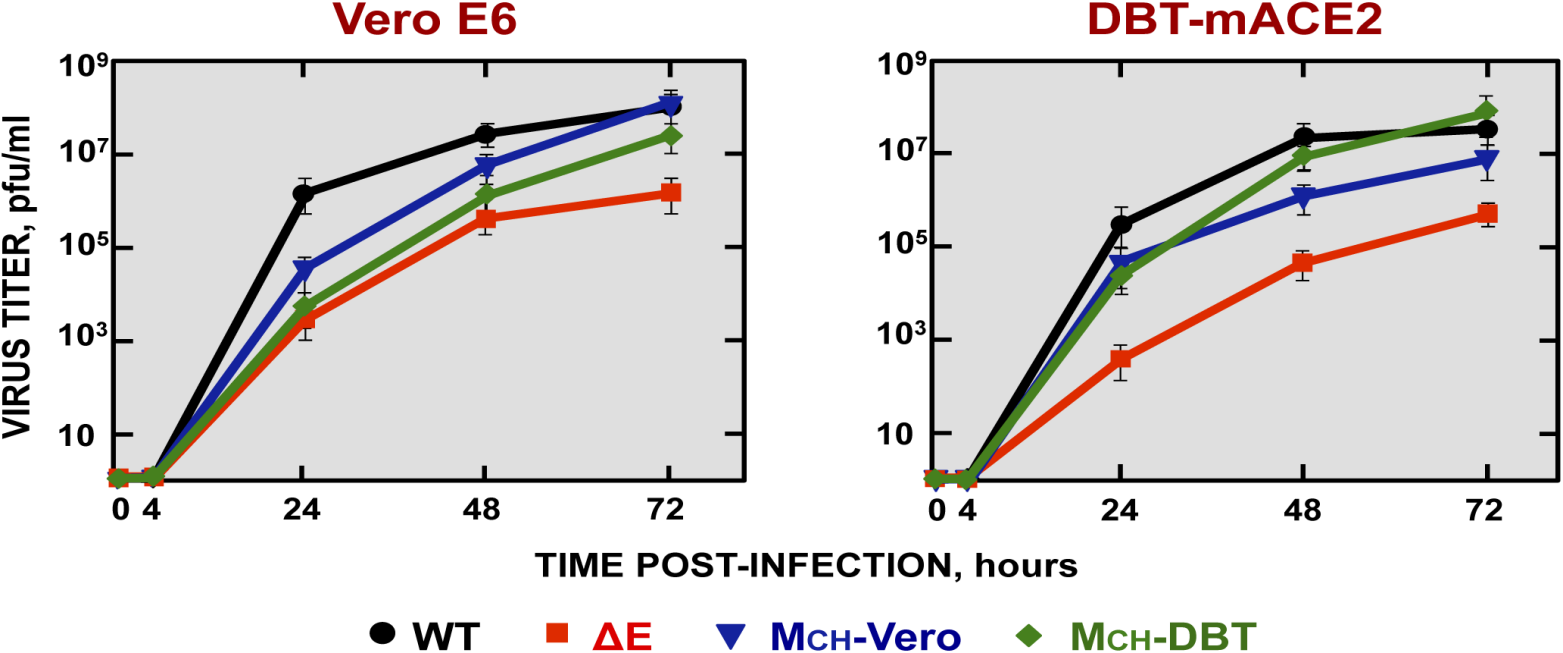
Growth kinetics of SARS-CoV-∆E viruses containing chimeric proteins. Subconfluent monolayers of Vero E6 and DBT-mACE2 cells were infected with wt, ΔE, MCH-Vero and MCH-DBT viruses at a moi of 0.001. Culture supernatants collected at 4, 24, 48 and 72 hpi were titrated by plaque assay.

To test whether the presence of MCH genes conferred a replication advantage to SARS-CoV-∆E *in vitro*, the growth kinetics of SARS-CoV-∆E-MCH-Vero (MCH-Vero) and SARS-CoV-∆E-MCH-DBT (MCH-DBT) were analyzed. Vero E6 and DBT-mACE2 cells were infected with the recombinant viruses (moi of 0.001) and viral titers were determined at the indicated hpi (Fig 2). MCH-Vero and M_CH_-DBT viruses showed lower titers at 24 hpi in Vero E6 cells compared to rSARS-CoV but both virus titers of the two chimeric viruses and rSARS-CoV were similar at 72 hpi. In contrast, a 100-fold decrease in viral growth was observed in rSARS-CoV-∆E-infected cells (Fig 2). Interestingly, chimeric proteins seemed to be specific for each cell type, as a ∆E virus containing a chimeric protein generated in Vero E6 cells (MCH-Vero) grew better in this cell line than in DBT-mACE cells, and the MCH-DBT virus generated in DBT-mACE2 cells specifically enhanced its growth in this cell line. This result indicated that the MCH protein provided a growth advantage for the virus, which partly compensated for the lack of the E protein.

### Virulence and viral growth of SARS-CoV-∆E-MCH in infected mice

**Fig 3:**
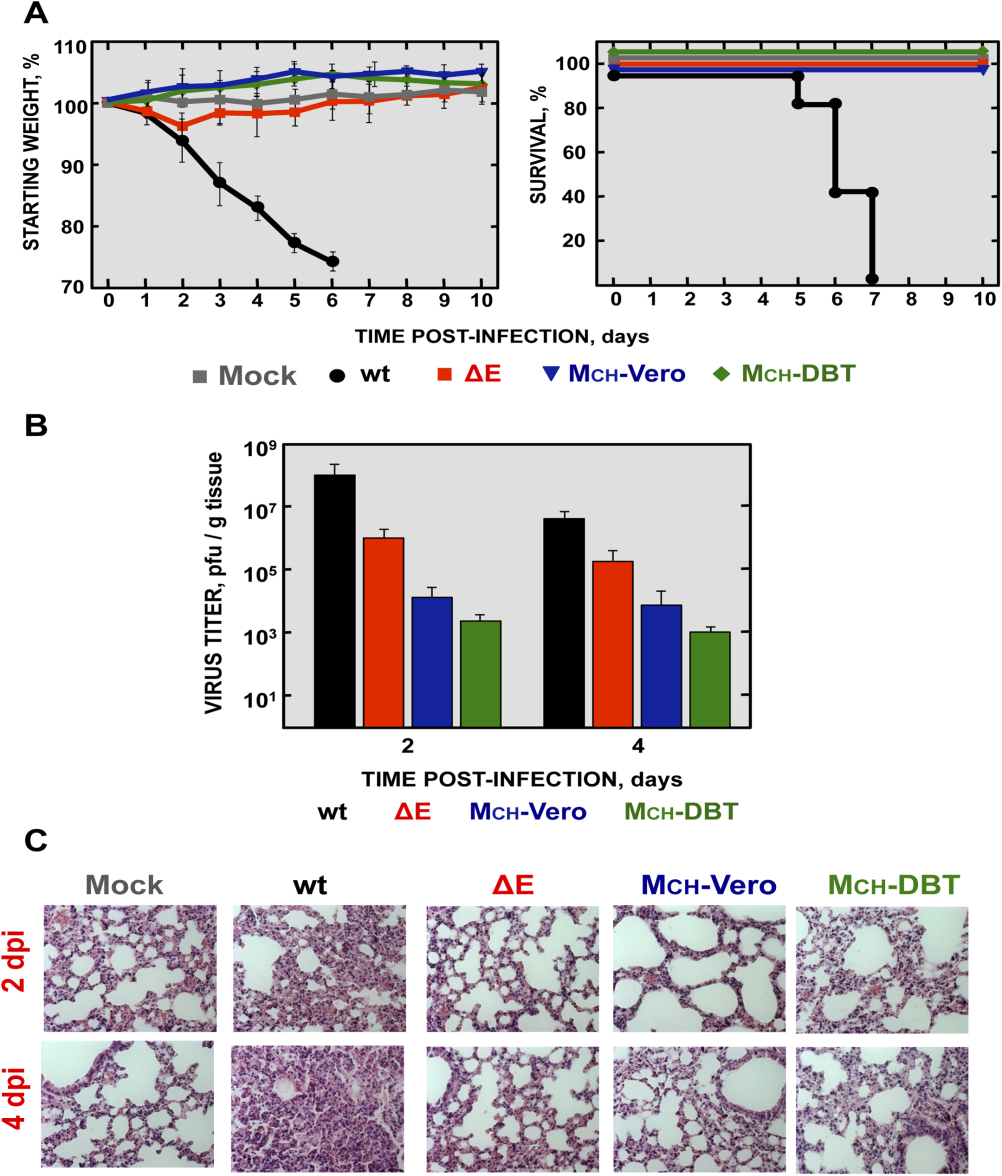
Virulence and viral growth of SARS-CoV-∆E after serial passage in cell culture. 16-week-old BALB/c mice were intranasally inoculated with 100,000 pfu of wt, ΔE, MCH-Vero and MCH-DBT viruses. (A) Weight loss and survival were monitored for 10 days. Data represent two independent experiments with 5 mice per group. (B) Viral titer in lungs was determined at 2 and 4 days post infection (n = 3, each day). Error bars represent standard deviations. (C) Lung tissue sections from mice infected with the different recombinant viruses were prepared and stained with hematoxylin and eosin at 2 and 4 dpi. Three independent mice per group were analyzed. Original magnification was 20x and representative images are shown.

To determine the effect of the MCH protein in pathogenesis, BALB/c mice were intranasally infected with the recombinant viruses using 100,000 pfu, and weight loss and survival were monitored for 10 days (Fig 3A). Mice infected with the parental virus (wt) showed signs of clinical disease at 2 days post infection (dpi), reflected by ruffled fur, shaking, loss of mobility and weight loss, resulting in the death of all mice at 6 dpi (Fig 3A). In contrast, mock-infected mice or mice infected with the ∆E virus, independently of whether the chimeric proteins (∆E, MCH-Vero and MCH-DBT) were present or absent, did not lose weight and all of them survived without symptoms of disease (Fig 3A).

To analyze the effect of the MCH protein on virus growth *in vivo*, BALB/c mice were intranasally inoculated with the recombinant viruses and euthanized at 2 and 4 dpi. Virus titers in the lungs were determined (Fig 3B). Viruses lacking the E protein, in the presence or absence of MCH protein, grew to lower titers in lungs at 2 and 4 dpi, as compared with those observed in mice infected with rSARS-CoV. Notably, rSARS-CoV-∆E replicated in the lung to higher levels than those containing the chimeric proteins (MCH-Vero and MCH-DBT) at both days p.i. Chimeric proteins only increased fitness in a cell type-dependent manner, i.e., viruses with the chimeric protein only grew better in the cell system in which this chimeric protein was generated. The virus containing a chimeric protein generated in Vero E6 cells grew better in this cell line, and the virus containing a chimeric protein generated in DBT-mACE2 cells showed an increased growth in these cells (Fig 2).

Lungs of mice infected with rSARS-CoV were highly edematous and showed profuse hemorrhagic areas at 2 and especially at 4 dpi (S2A Fig), leading to a significant increase in lung weight at 4 dpi (S2B Fig). Lung sections from mock-infected mice or mice infected with rSARS-CoV-∆E, MCH-Vero and MCH-DBT (Fig 3C) showed minimal damage at 2 and 4 dpi. In contrast, analysis of the lungs of mice infected with rSARS-CoV revealed extensive inflammatory cell infiltration and edema in alveolar and bronchiolar airways (Fig 3C).

### Identification of E protein domains restored by the generation of chimeric proteins

**Fig 4:**
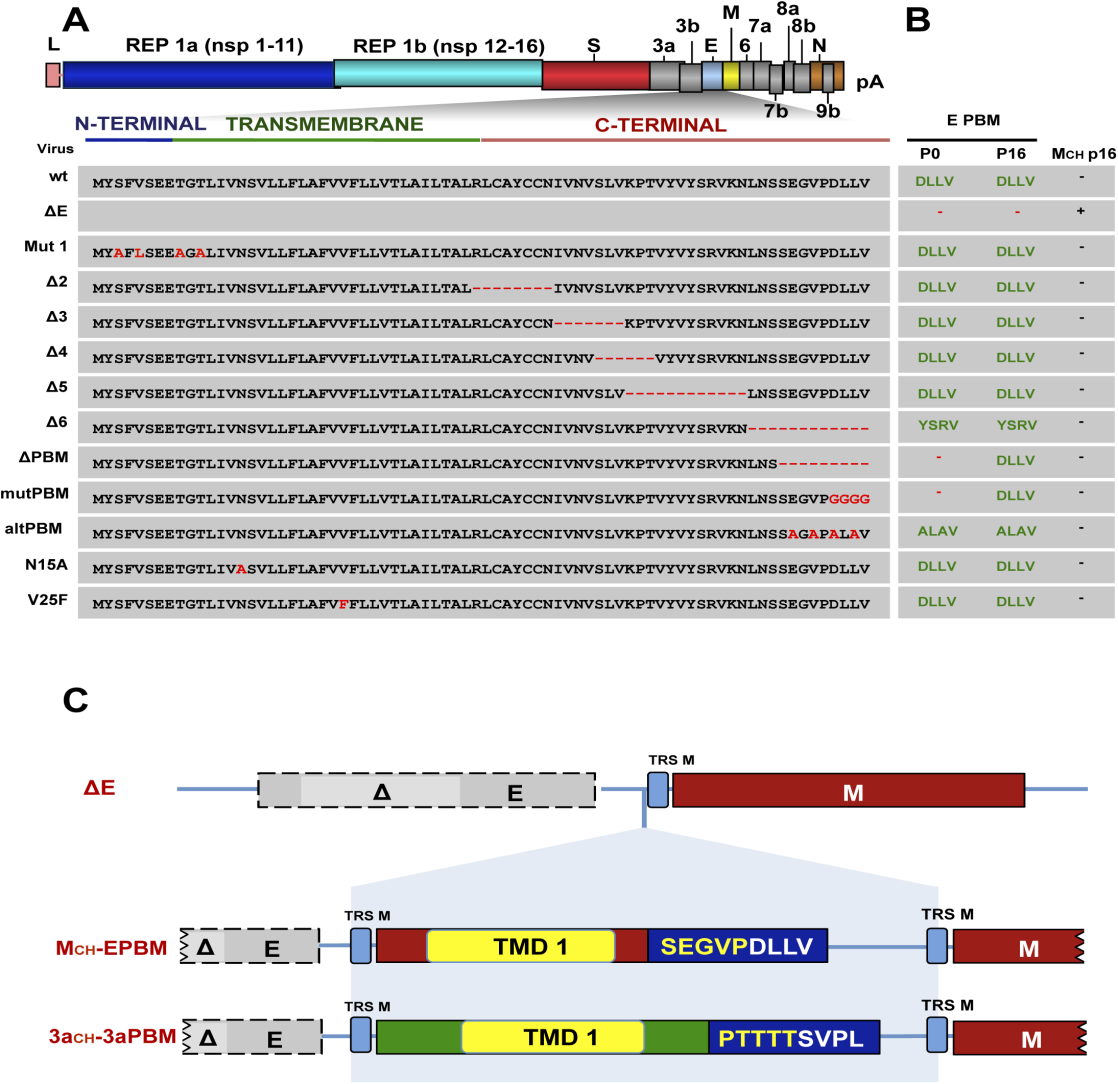
Identification of E protein domain involved in chimeric protein generation in cell culture. (A) The SARS-CoV genome is shown at the top, and the expanded region shows the E protein sequence and its different regions. Grey boxes indicate a set of recombinant SARS-CoVs including mutations or deletions in different regions of E protein. Mutations and deletions are shown in red. Recombinant viruses were passaged 16 times in Vero E6 cells and the presence of chimeric genes and E protein PBMs was analyzed by sequencing, using specific primers. (B) Schematic indicating the presence and sequence (green) or absence (red) of PDZ-binding motifs at the end of the different E proteins (E PBM) at passages 0 (p0) and 16 (p16). Presence or absence of chimeric proteins after 16 serial passages in cell culture are indicated by (–) and (+), respectively. (C) Representation of recombinant viruses generated of SARS-CoV-∆E-MCH-EPBM (MCH-EPBM) and SARS-CoV∆E-3aCH-3aPBM (3aCH-3aPBM). Top, ∆E represents the genomic sequence of viruses lacking E protein. Grey boxes indicate E gene with a partial deletion highlighted in light grey (∆). Transcription-regulating sequences (TRSs) of the different genes are shown in blue boxes and the membrane gene (M) is shown in red. TMD1 represents the transmembrane domain of the indicated proteins. DLLV and SVPL represent the last four amino acids forming different PDZ-binding motifs.

The chimeric protein MCH was generated after rSARS-CoV-∆E passage in cell culture, but not when full-length E protein was present, suggesting that it compensated for functions originally performed by E protein. To identify such E protein functional domains, a set of recombinant SARS-CoVs (Fig 4A), with mutations or deletions in different regions of E protein [23, 24, 53], was passaged 16 times in Vero E6 cells (Fig 4). In Mut 1, several amino acid substitutions were introduced at the E protein amino-terminal region. rSARS-CoV deletion mutants ∆2, ∆3, ∆4, ∆5 and ∆6 included sequential or partially overlapping small deletions of 6 to 12 amino acids in the carboxy-terminus of E protein. Interestingly, the last 6 amino acids within the Δ6 virus (YSRVKN; Fig 4) revealed an alternative PBM at the carboxy-terminal domain of the protein [53]. In recombinant ∆PBM, the last 9 amino acids of E protein were deleted, truncating the carboxy-terminus and eliminating the E protein PBM. In mutPBM, the PBM was abolished by mutating the last 4 amino acids to glycine, maintaining the full-length E protein. In contrast, in altPBM, 4 amino acids within E protein carboxy-terminal region were mutated to alanine, maintaining an active PBM domain [24]. In SARS-CoV N15A and V25F mutants, ion channel activity of E protein was abolished by one point mutation in the transmembrane domain (Fig 4A). Vero E6 cells were infected with each of the recombinant viruses at a moi of 0.5 and supernatants were serially passaged for 16 times, and the presence of MCH gene was determined by sequence analysis. The results indicated that the MCH was not generated when the E protein contained a PBM sequence (rSARS-CoV, Mut 1, ∆2, ∆3, ∆4, ∆5, ∆6, altPBM, N15A and V25F) (Fig 4B). When the PBM was absent (∆PBM and mutPBM), a new PBM containing the original sequence was added to the carboxy-terminal end of mutated E protein in all cases, reinforcing the importance of the PBM domain during infection. A virus incorporating the chimeric MCH protein was only generated when E protein was completely deleted (∆E), i.e., when the restoration of a PBM on the E protein was not possible.

To further analyze whether SARS-CoV requires a transmembrane protein containing a PBM, we generated two recombinant rSARS-CoV-∆E that contained artificial chimeric proteins (Fig 4C). In SARS-CoV-∆E-MCH-EPBM (MCH-EPBM), a chimeric protein containing the first transmembrane domain of the M protein fused to the last nine amino acids of E protein, encompassing the PBM, was introduced. In SARS-CoV-∆E-3aCH-3aPBM (3aCH-3aPBM), the chimeric protein was formed by the first transmembrane domain of 3a protein and a PBM composed by the last nine amino acids of 3a protein (Fig 4C). Both viruses were passaged 16 times in Vero E6 cells and compensatory mutations were not detected after sequencing. All these data indicated that the virus requires a transmembrane protein displaying a PBM and that novel proteins with a PBM compensate for the loss of the E protein PBM.

### Incorporation of a PBM domain after passage in the mouse lung

**Fig 5:**
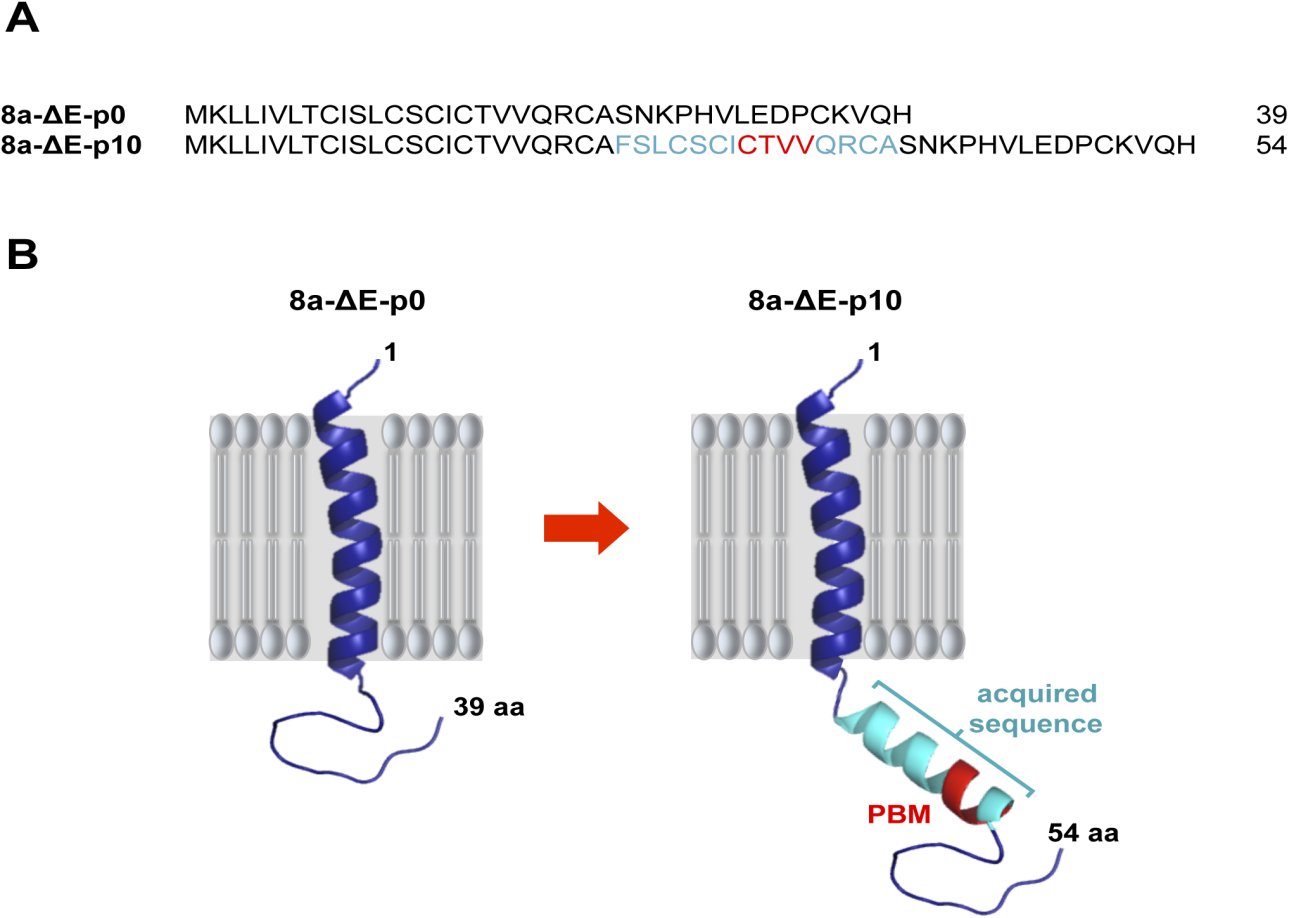
Stability of SARS-CoV-∆E after serial passage in mice. A recombinant virus lacking the E protein (SARS-CoV-∆E) was passaged in BALB/c mice using an initial moi of 100,000 pfu. After 10 serial passages the last third of the genome was sequenced using specific primers. (A) Sequence corresponding to native 8a protein (8a) and to mutated protein generated after passage in mice (8a-dup). New incorporated amino acids are highlighted in light blue. (B) Predicted structures for 8a protein at passage 0 (8a) and 10 (8a-dup) in mice. Presence of new incorporated amino acids in 8a-dup and PBM are highlighted in light blue and red, respectively.

**Fig 6:**
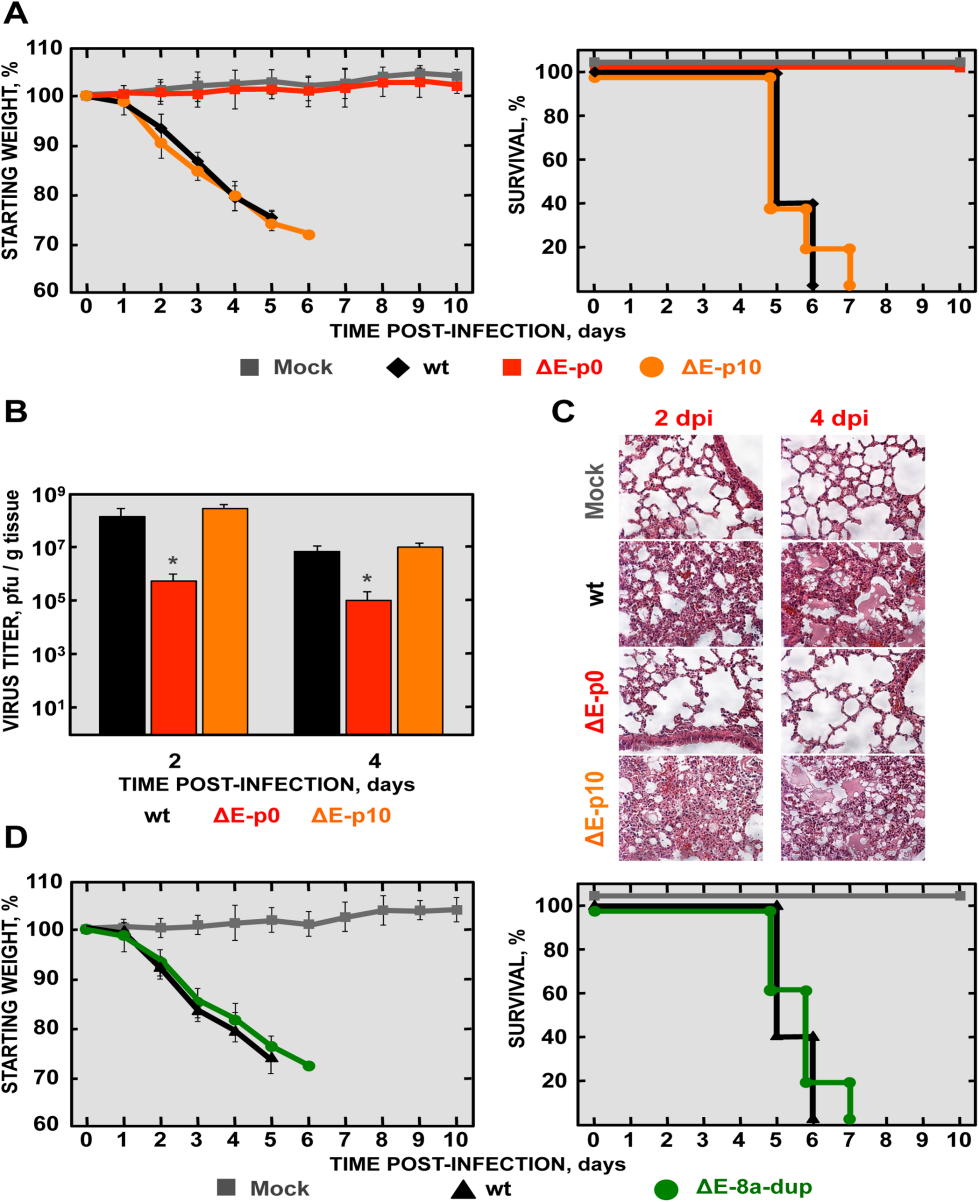
Virulence and viral growth of SARS-CoV-∆E after serial passage in mice. 16-week-old BALB/c mice were intranasally inoculated with 100,000 pfu of wt, ΔE and ∆E-8a-dup viruses. (A) Weight loss and survival were monitored for 10 days. Data represent two independent experiments with 5 mice per group. (B) Viral titer in lungs was determined at 2 and 4 days post infection (n = 3, each day). Error bars represent standard deviations. Statistically significant data are indicated with one asterisk (*P* < 0.05). (C) Lung tissue sections from mice infected with the different recombinant viruses were prepared and stained with hematoxylin and eosin at 2 and 4 dpi. Three independent mice per group were analyzed. Original magnification was 20x and representative images are shown. (D) 16-week-old BALB/c mice were intranasally inoculated with 100,000 pfu of wt or ΔE-8a-dup viruses and were monitored daily for weight loss and survival.

MCH-Vero and MCH-DBT exhibited an attenuated phenotype (see above). To analyze the genetic stability of recombinant SARS-CoV-∆E in BALB/c mice, virus was passaged every 48 hours by intranasal inoculation. A partial duplication of 45 nucleotides was found within 8a gene (Fig 5), leading to the incorporation of a fragment of 15 amino acids at the carboxy-terminus of 8a protein, and generating a novel 8a protein (8a-dup) with an internal PBM (CTVV) (Fig 5A and 5B). To determine the virulence of this novel virus (SARS-CoV-∆E-8a-dup), we infected a new cohort of BALB/c mice and assessed survival and weight loss, and measured virus titers. Mice infected with SARS-CoV-∆E did not lose weight and all survived. In contrast, SARS-CoV-∆E-8a-dup grew to titers similar to those of rSARS-CoV and developed profound weight loss, developing signs of illness and death by 7 dpi (Fig 6A and 6B). Histological examination of lungs from SARS-CoV-∆E-infected mice showed absence of lung damage at both dpi. In contrast, lungs of mice infected with rSARS-CoV or SARS-CoV-∆E-8a-dup showed substantial perivascular, peribronchial and interstitial cellular infiltration and edema at 2 and 4 dpi (Fig 6C). To further confirm the relevance of the partial duplication within 8a gene in the induction of virulence, a recombinant SARS-CoV-∆E with an 8a-dup gene was generated (rSARS-CoV-∆E-8a-dup). Virulence during infection with rSARS-CoV-∆E-8a-dup was evaluated as described above (Fig 6D). The engineered virus (rSARS-CoV-∆E-8a-dup) was as virulent as the SARS-CoV-∆E-8a-dup generated after SARS-CoV-∆E passage *in vivo* (Fig 6D). These results indicated that SARS-CoV-∆E regained virulence after serial passage in mice.

### Mechanisms involving SARS-CoV-∆E-8a-dup reversion to virulence

**Fig 7:**
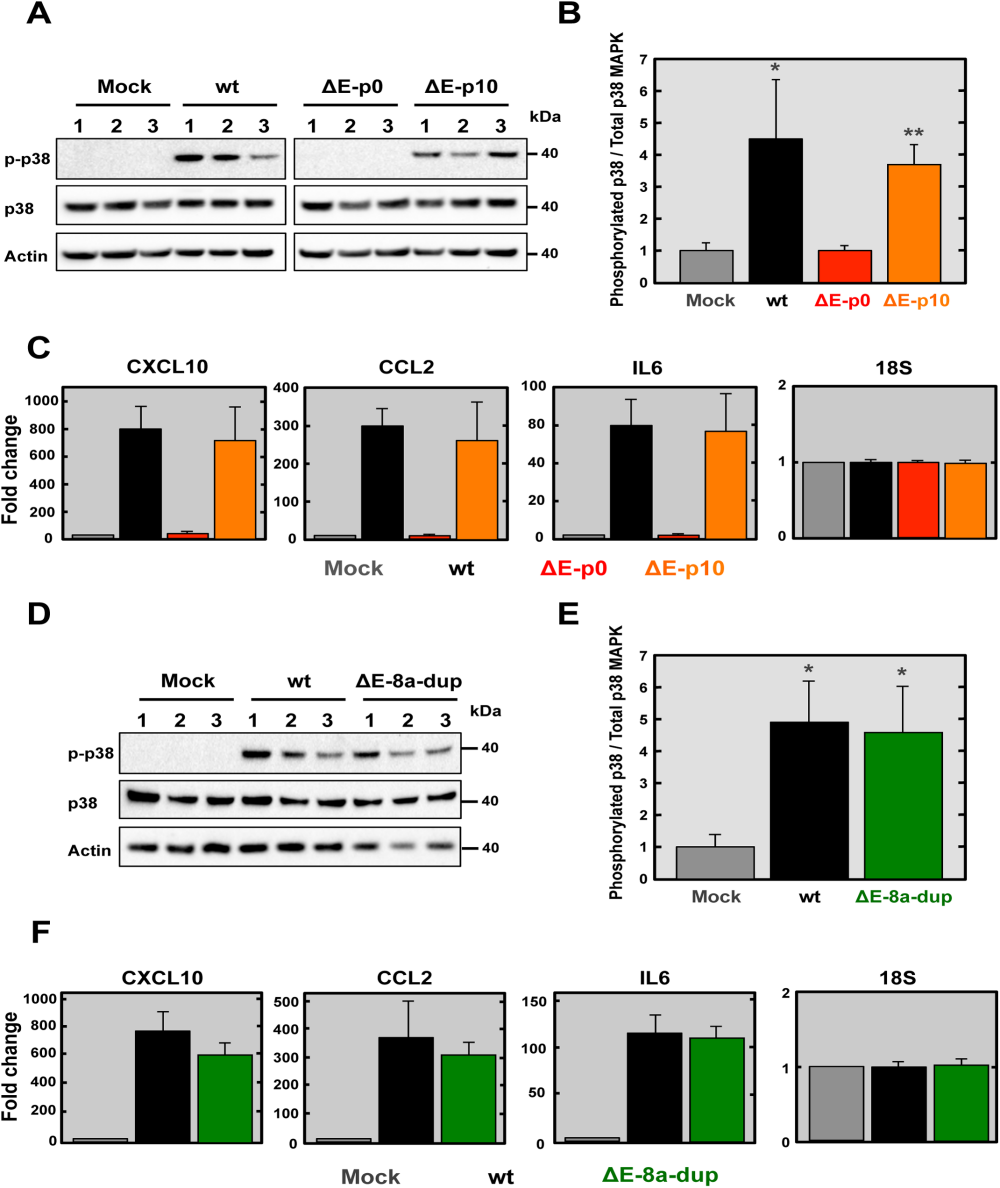
Analysis of p38 MAPK activation and inflammatory cytokines expression during infection with recombinant SARS-CoV-∆E virus after passage in mice. (A) Vero E6 cells were mock-infected or infected with the wt, ∆E and ∆E-8a-dup viruses at a moi of 0.3 and the presence of active phosphorylated (p-p38) and total (p38) p38 MAPK was detected by Western blot analysis at 24 hpi. Actin was used as control. Lines 1, 2 and 3 indicated three independent experiments analyzed in each case. (B) Phospho and total p38 MAPK amounts were quantified by densitometric analysis. The graph shows the phosphorylated p38/total p38 MAPK ratio in Vero E6 cells infected with wt, ΔE and ∆E-8a-dup viruses at 24 hpi. Statistically significant data compared to mock-infected cells are indicated with one (P < 0.05) or two (P < 0.01) asterisks. (C) Expression of inflammatory cytokines in lungs of infected mice evaluated by RT-qPCR at 2 dpi. Three independent experiments were analyzed with similar results in all cases. Error bars represent the means of three experiments analyzed for each condition. (D) Vero E6 cells were mock-infected or infected with the wt or ∆E-8a-dup viruses at a moi of 0.3 and the presence of active phosphorylated (p-p38) and total (p38) p38 MAPK was detected by Western blot analysis at 24 hpi. Actin was used as control. Lines 1, 2 and 3 indicated three independent experiments analyzed in each case. (E) Phospho and total p38 MAPK amounts were quantified by densitometric analysis. The graph shows the phosphorylated p38/total p38 MAPK ratio in Vero E6 cells infected with wt and ∆E-8a-dup viruses at 24 hpi. Statistically significant data compared to mock-infected cells are indicated with one (P < 0.05) asterisk. (F) Expression of inflammatory cytokines in lungs of infected mice evaluated by RT-qPCR at 2 dpi. Three independent experiments were analyzed with similar results in all cases. Error bars represent the means of three experiments analyzed for each condition.

SARS-CoV infection is associated with p38 mitogen-activated protein kinase (MAPK) activation and elevated levels of pro-inflammatory cytokines [24, 54–56]. To begin to determine the basis of increased virulence exhibited by SARS-CoV-∆E-8a-dup, p38 MAPK activation and pro-inflammatory cytokine expression were analyzed during infection (Fig 7). p38 MAPK activation was analyzed by Western blot at 24 hpi using a phospho-p38 MAPK (p-p38) specific antibody. Antibodies recognizing the total endogenous p38 MAPK and actin were used as controls. A significant increase in p38 MAPK activation, assessed at 24 hpi using a phospho-p38 MAPK (p-p38)-specific antibody and Western blot analysis, was observed in SARS-CoV-∆E-8a-dup-infected compared to SARS-CoV-∆E or mock-infected cells (Fig 7A and 7B).

To test whether pro-inflammatory cytokine expression was induced during SARS-CoV-∆E-8a-dup infection, we analyzed the expression of several genes previously associated with SARS-CoV pathology [24, 57] including: chemokine (C-X-C motif) ligand 10 (CXCL10), chemokine (C-C motif) ligand 2 (CCL2) and interleukin 6 (IL6) (S3 Table). 18S ribosomal RNA was used to normalize the data, as previously described [58, 59]. BALB/c mice were mock-infected or infected with 100,000 pfu of recombinant SARS-CoV, and lungs were collected at 2 dpi. A significant increase in the expression of all pro-inflammatory cytokines in the lungs was observed during infection with virulent viruses (rSARS-CoV and SARS-CoV-∆E-8a-dup) (Fig 7C). In contrast, infection with the recombinant virus lacking E protein at passage 0 (SARS-CoV-∆E) did not induce the expression of pro-inflammatory cytokines. Activation of p38 MAPK and pro-inflammatory cytokines expression during infection with rSARS-CoV-∆E-8a-dup were evaluated as described above. The engineered virus (rSARS-CoV-∆E-8a-dup) induced similar p38 MAPK activation (Fig 7D and 7E) and overexpression of proinflammatory cytokines (Fig 7F) as compared with the SARS-CoV-∆E-8a-dup generated after SARS-CoV-∆E passage *in vivo*. These data indicated that reversion of SARS-CoV-∆E-8a-dup to a virulent phenotype was associated with an exacerbated immune response similar to that triggered during infection with the rSARS-CoV.

### Generation of recombinant SARS-CoV-nsp1* viruses

**Fig 8:**
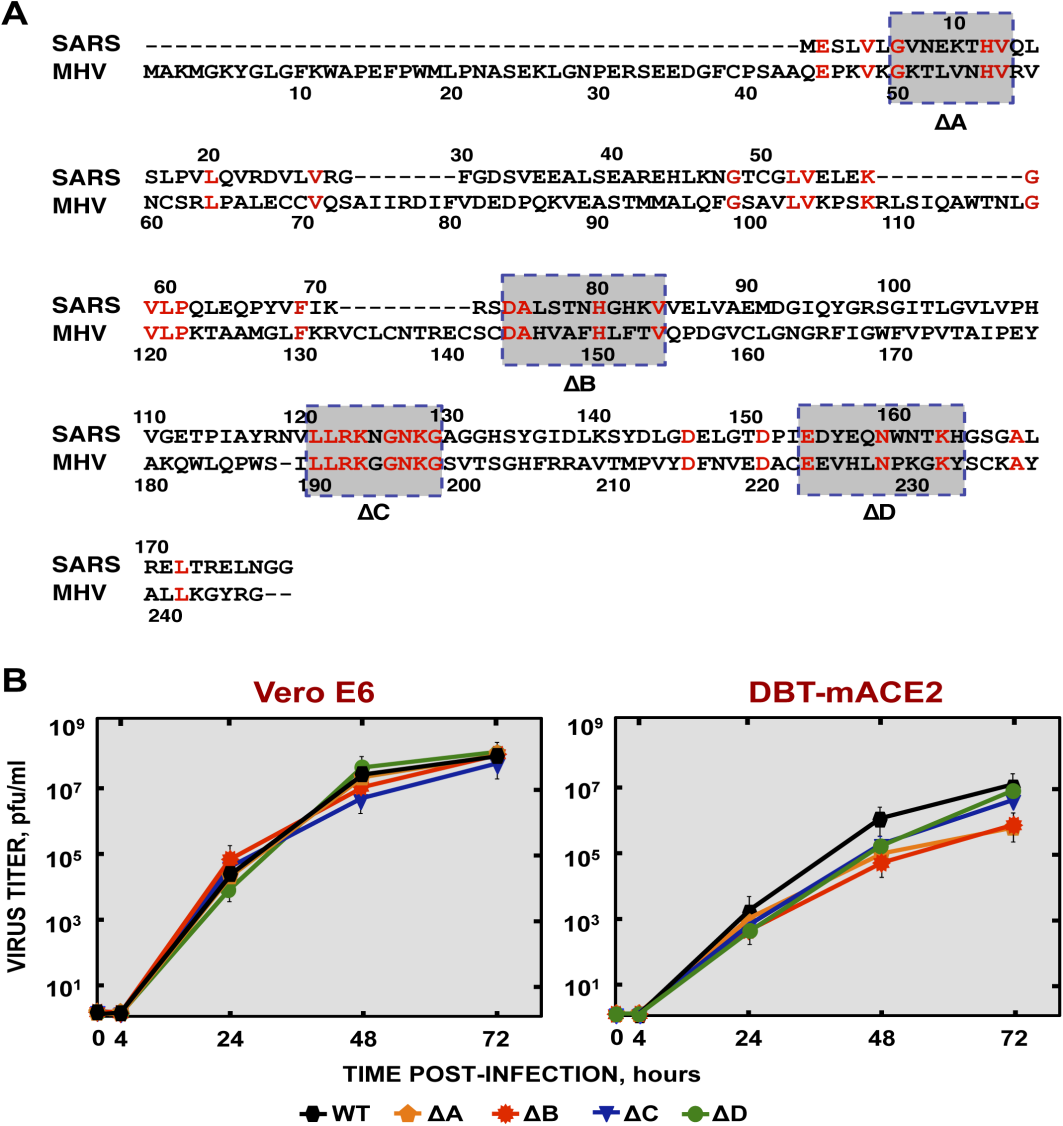
Generation and growth kinetics of SARS-CoVs lacking small regions within the nsp1 protein. (A) Sequence alignment of nsp1 from SARS-CoV and MHV. The conserved amino acids in these proteins are indicated in red. Gray boxes represent the amino acids deleted within the nsp1 protein in ΔA, ΔB, ΔC and ΔD viruses. (B) Mutant viruses growth kinetics. Subconfluent monolayers of Vero E6 and DBT-mACE2 cells were infected with wt or SARS-CoV-nsp1* viruses at a moi of 0.001. At different times post infection, virus titers were determined by plaque assay on Vero E6 cells. Error bars represent standard deviations of the mean of three independent experiments.

SARS-CoV-ΔE reverted to a virulent phenotype after serial passages in mice. To increase the genetic stability of the vaccine candidate, we introduced small attenuating deletions in the E gene, instead of deleting of the full-length E protein [53] as described above. In addition, to increase vaccine biosafety, we introduced additional attenuating mutations within the SARS-CoV nsp1 gene. To identify domains within the SARS-CoV nsp1 protein that could contribute to virulence, we compared the sequence to that of the MHV nsp1 (Fig 8A), with the goal of identifying conserved regions that could be functionally important. Based on this information, four mutant viruses (rSARS-CoV-nsp1*) were generated by introducing deletions of 8 to 11 amino acids into the nsp1 protein (rSARS-CoV-nsp1-∆A, -∆B, -∆C and -∆D Fig 8A). All the deletion mutants grew to similar titers as rSARS-CoV in Vero E6 cells (Fig 8B). However the ∆A and ∆B mutants grew to lower titers in DBT-mACE2 cells.

### Pathogenicity of SARS-CoV-nsp1* viruses in mice

**Fig 9:**
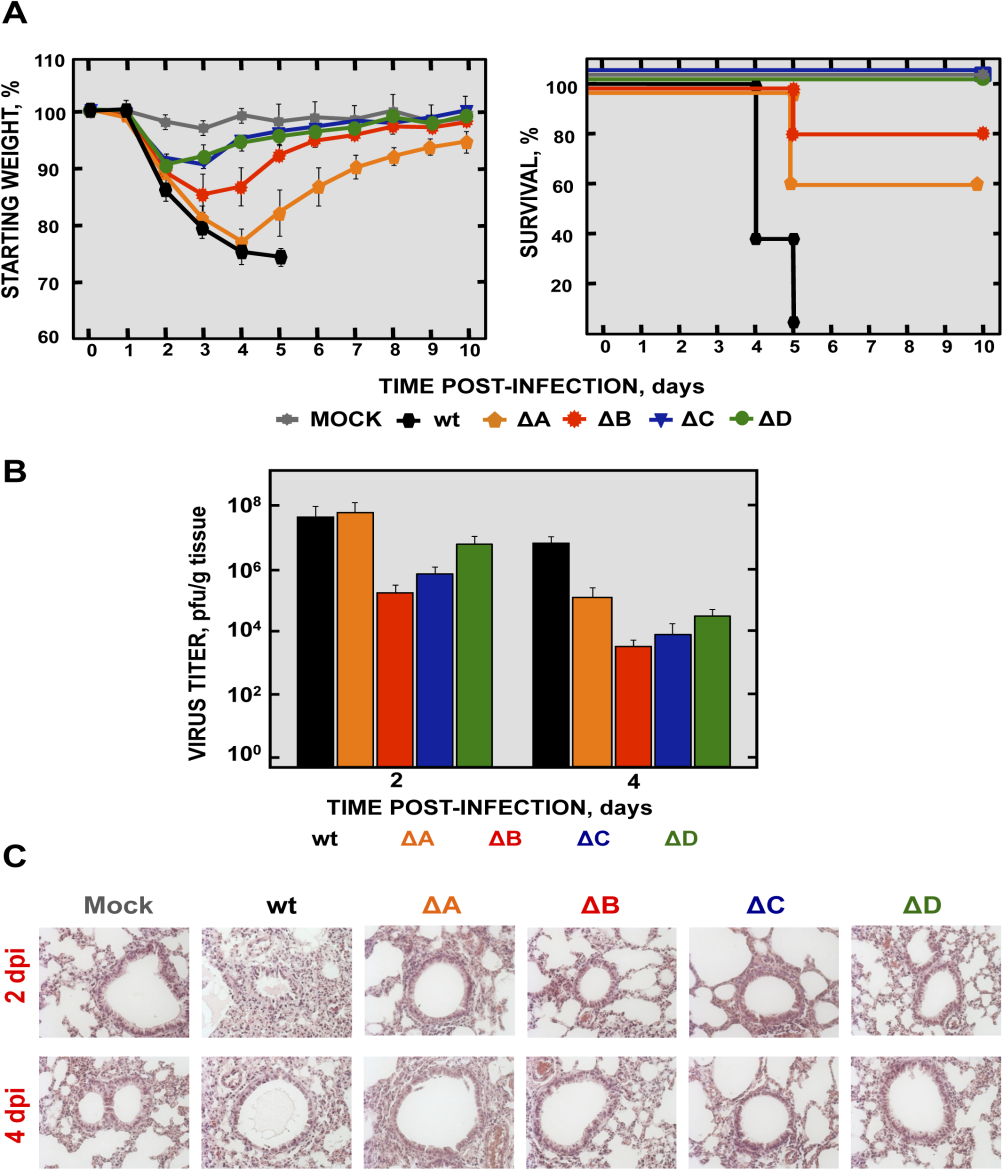
Virulence and viral growth of SARS-CoV-nsp1* mutants *in vivo*. BALB/c mice were intranasally infected with 100,000 pfu of wt, and SARS-CoV-nsp1-ΔA, -ΔB, -ΔC and -ΔD viruses (5 mice per group). (A) Animals were monitored daily for weight loss and survival. (B) Viral titers in lungs were determined at 2 and 4 days post infection (3 mice per group and time point). Error bars represent the standard deviations from three independent mice in each case. (C) Lung tissue sections from mice infected with the different recombinant viruses were prepared and stained with hematoxylin and eosin at 2 and 4 dpi. Three independent mice per group were analyzed. Original magnification was 20x and representative images are shown.

To analyze the effects of these deletions *in vivo*, mice were intranasally infected with mutants rSARS-CoV-nsp1-∆A, -∆B, -∆C and -∆D, and daily monitored for 10 days. SARS-CoV-nsp1-∆C and -∆D infected mice transiently lost a small amount of weight and all mice survived. In contrast, mice infected with SARS-CoV lost weight, and all died by day 5 (Fig 9A). Mice infected with rSARS-CoV-nsp1-∆A or rSARS-CoV-nsp1-∆B lost 20 and 15% of their initial weight by day 3, with survival reduced to 60 and 80%, respectively (Fig 9A). These data indicated that deletion of regions C and D within nsp1 protein led to attenuated mutants, whereas deletion of regions A and B were only partially attenuating. With the exception of rSARS-CoV-nsp1-∆A at day 2 p.i., virus titers were reduced compared to rSARS-CoV-infected mice (Fig 9B). There was not a strict correlation between virus titers and virulence, possibly because nsp1 is involved in the countering IFN production after infection.

No significant changes on gross inspection of lungs or in their weight were observed when the lungs of mock-infected and SARS-CoV-nsp1-∆C and -∆D-infected mice were compared (Figs 9C and S3). In contrast, lungs from mice infected with the rSARS-CoV and, to a much lower extent SARS-CoV-nsp1-∆A and -∆B-infected mice lungs, showed evidence of hemorrhage (S3 Fig). In addition, rSARS-CoV-infected mice showed lung weight increase, not observed with the lungs of SARS-CoV-nsp1*-infected mice. Compared to mock-infected mice, lungs from rSARS-CoV-infected mice showed clear inflammatory infiltrates and alveolar and bronchiolar edema (Fig 9C). In contrast, mice infected with the viruses rSARS-CoV-nsp1* showed no (SARS-CoV-nsp1-∆C and -∆D), or minimal (SARS-CoV-nsp1-∆A and -∆B) lung damage. These data correlated well with the virulence observed for the SARS-CoV-nsp1* mutants, showing that the most attenuated viruses were those that induced less lung pathology *in vivo*.

**Fig 10:**
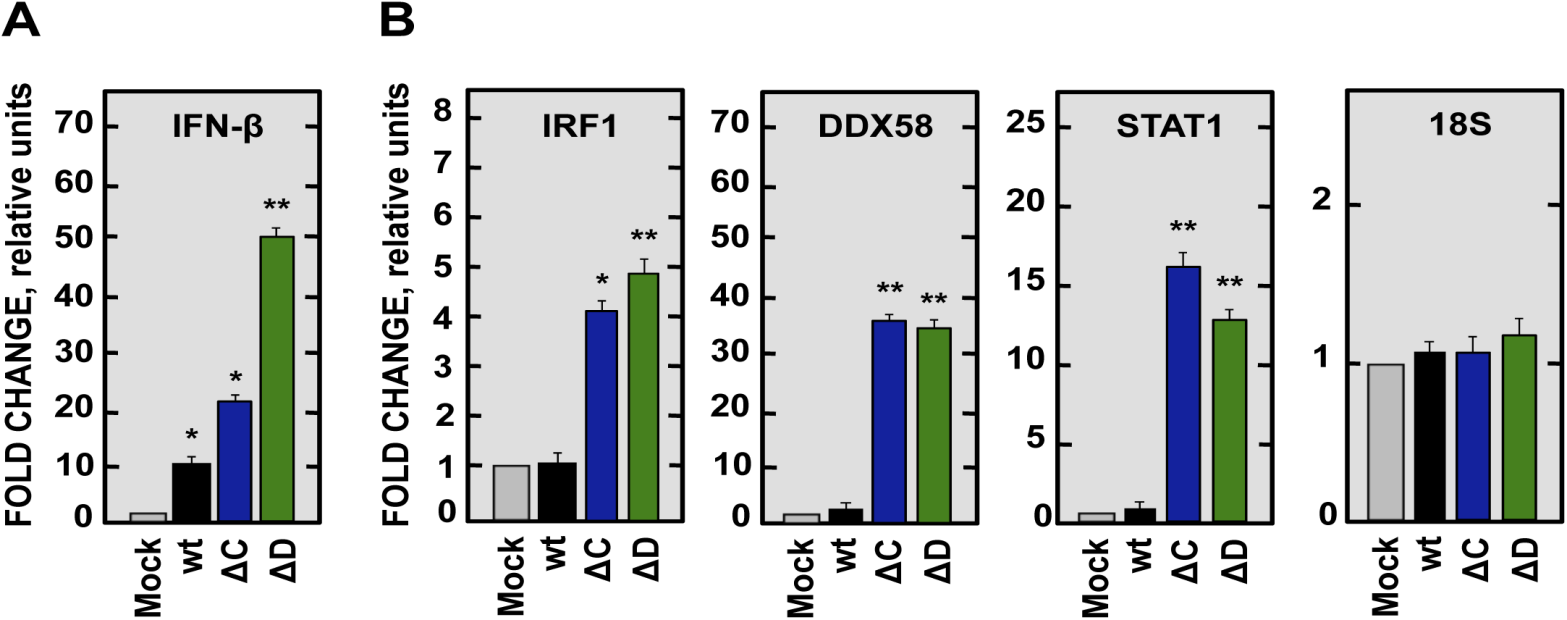
Expression of IFN-β and ISGs in SARS-CoV-nsp1* attenuated mutants infected cells. DBT-mACE2 cells were mock-infected or infected at a moi of 0.125 with SARS-CoV-nsp1* attenuated mutants or SARS-CoV (wt). Cellular RNAs were extracted at 48 hpi and the expression of the indicated genes was determined by RT-qPCR. In each case, the corresponding mRNA expression levels were plotted as fold change relative to expression levels in uninfected cells. (A) IFN-β. (B) ISGs such as IRF1, DDX58 and STAT1, and 18S rRNA as a control. Error bars represent standard deviations of the means from three experiments. Statistically significant data compared to uninfected cells are indicated with one (*P* < 0.05) or two (*P* < 0.01) asterisks.

Since nsp1 has anti-interferon activity, we next measured expression of IFN and IFN-stimulated genes after infection with rSARS-CoV, rSARS-CoV-nsp1-∆C and -∆D. We focused on the ∆C and ∆D viruses, because these viruses were fully attenuated and had an efficient growth *in vivo*. SARS-CoV-nsp1-∆C and -∆D induced higher levels of IFN-β and ISGs (IRF1, DDX58, and STAT1), compared to mock-infected and rSARS-CoV-infected cells (Fig 10A and 10B). This effect was specific, as the expression of control 18S rRNA was the same in virus-infected cells or mock-infected cells (Fig 10B). These results indicated that deletion of regions C and D of nsp1 restored IFN responses, leading to virus attenuation.

### rSARS-CoV-nsp1-∆C and -∆D are potential vaccine candidates

**Fig 11:**
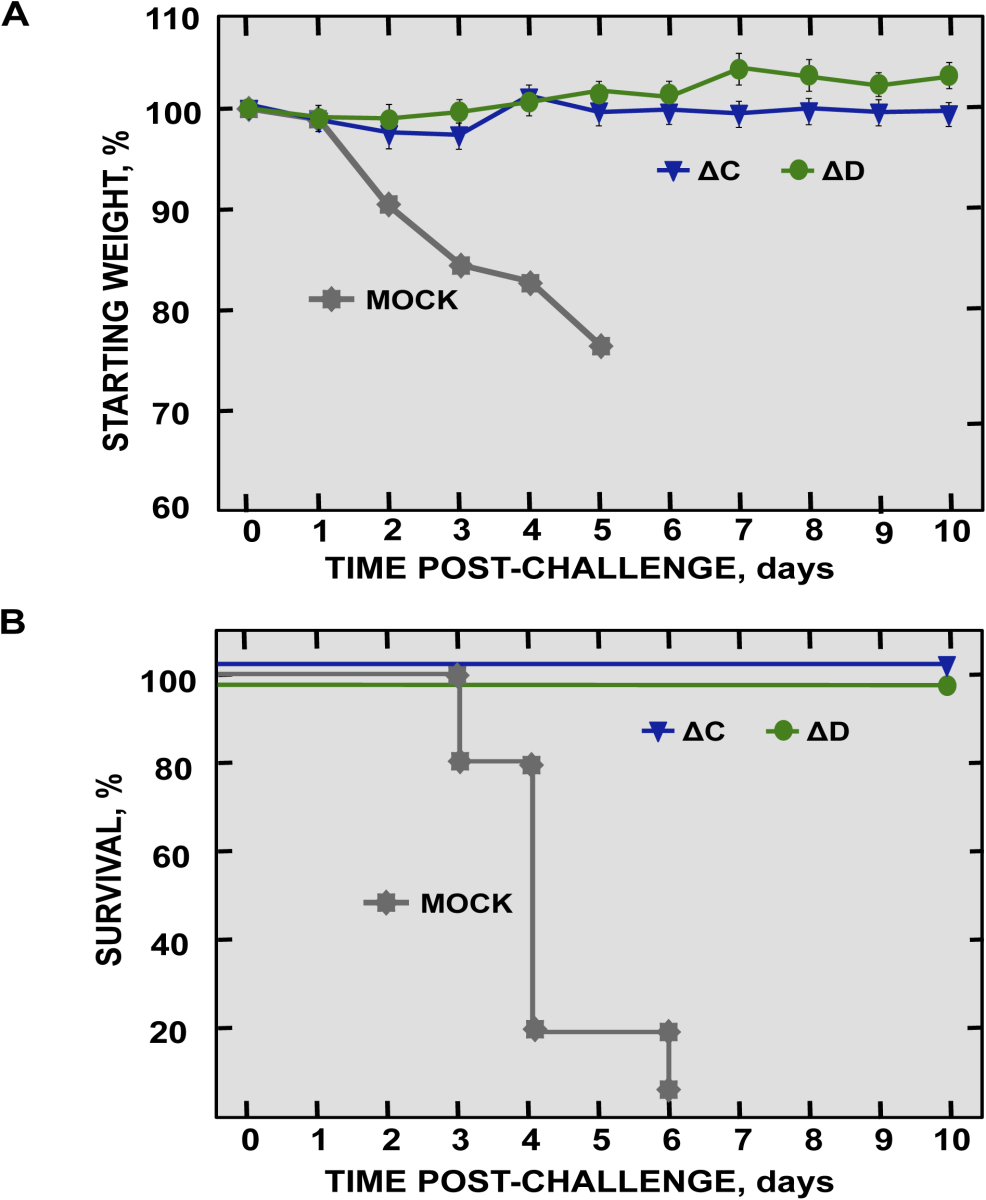
Protection conferred by immunization with SARS-CoV-nsp1* mutants. 16-week old BALB/c mice were mock-immunized or immunized with 6000 pfu of SARS-CoV-ΔC and -ΔD mutants, and challenged at day 21 post-immunization with 100,000 pfu of SARS-CoV-wt virus (5 mice per group). Weight loss (A) and survival (B) were recorded daily.

To analyze whether SARS-CoV-nsp1-∆C and -∆D induced protective immune responses, mice were intranasally vaccinated with SARS-CoV-nsp1-∆C and -∆D, and challenged 21 days later with rSARS-CoV. After challenge, mock-vaccinated mice rapidly lost weight, and all mice died by day 6 (Fig 11A and 11B). In contrast, mice immunized with SARS-CoV-nsp1-∆C and -∆D viruses, did not significantly lose weight, and 100% survived the challenge (Fig 11A and 11B).

### Generation of rSARS-CoV mutants with deletions in both nsp1 and E genes

**Fig 12:**
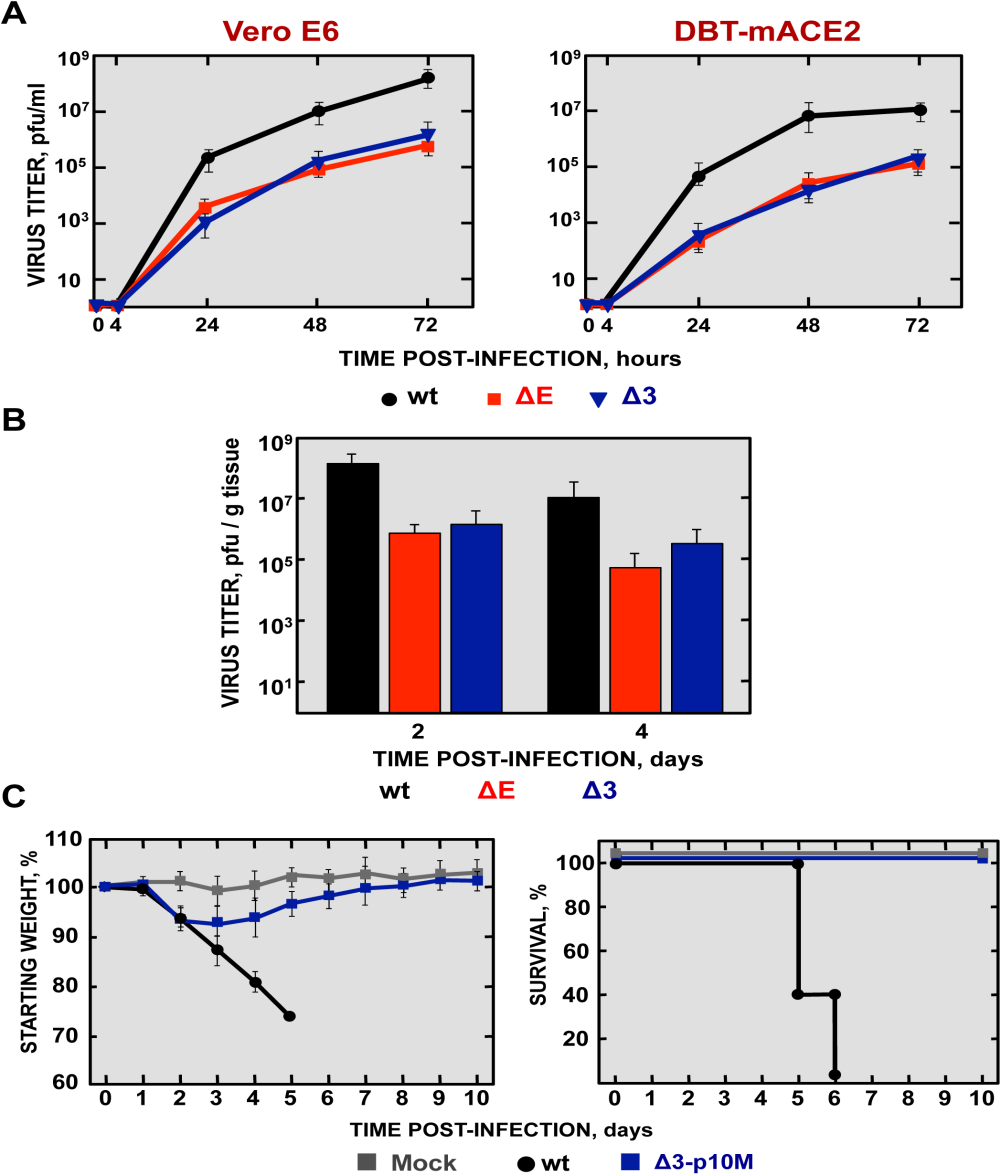
Viral growth of SARS-CoV-∆3 and virulence after serial passage in mice. (A) Subconfluent monolayers of Vero E6 and DBT-mACE2 cells were infected with wt, ΔE and Δ3 viruses at a moi of 0.001. Culture supernatants collected at 4, 24, 48 and 72 hpi were titrated by plaque assay. (B) 16-week-old BALB/c mice were intranasally inoculated with 100,000 pfu of wt, ΔE and Δ3 viruses. Viral titer in lungs was determined at 2 and 4 days post infection (n = 3, each day). Error bars represent standard deviations. (C) Weight loss and survival were monitored for 10 days (5 mice per group).

**Fig 13:**
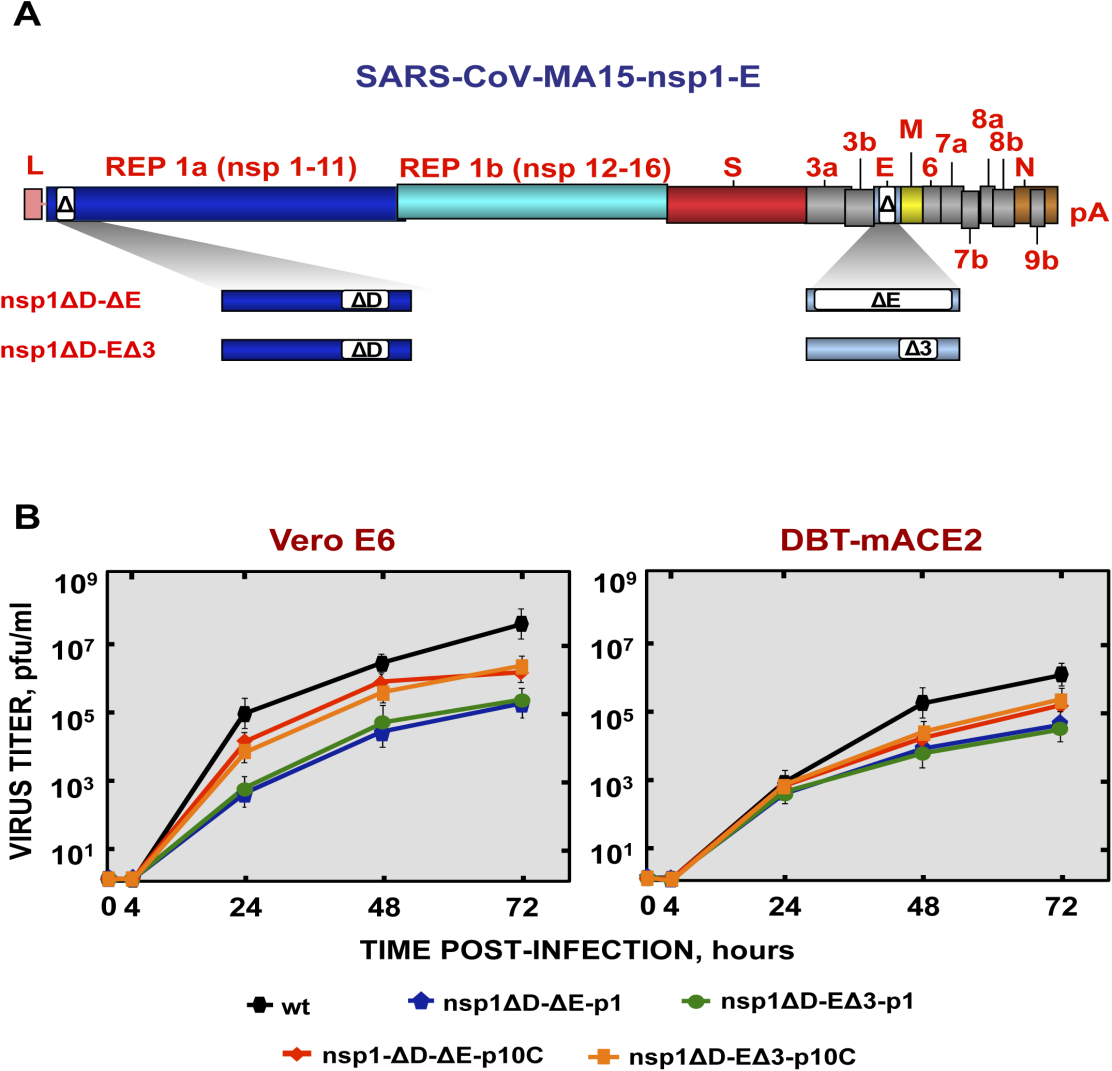
Generation and growth kinetics of SARS-CoV mutants with deletions in both nsp1 and E genes. (A) SARS-CoV genome is shown in the top, and the expanded region shows the nsp1 and E genes. White boxes represent the amino acids stretches deleted in both proteins in each virus. (B) Mutant viruses growth kinetics. Subconfluent monolayers of Vero E6 and DBT-mACE2 cells were infected with wt, SARS-CoV-nsp1ΔD-ΔE and SARS-CoV-nsp1ΔD-EΔ3 at passage 1 and 10 (-p1 and -p10C) viruses at a moi of 0.001. At different times post infection, virus titers were determined by plaque assay on Vero E6 cells. Error bars represent standard deviations of the mean using data from three independent experiments.

In order to develop a safe vaccine candidate, mutant viruses with deletions in both nsp1 and E genes were engineered. A rSARS-CoV deleted in the nsp1 D domain and the E protein (SARS-CoV-nsp1ΔD-ΔE), and a second mutant virus with deletions of the nsp1 D domain coupled with a small deletion (E∆3) (Fig 4A) in the E protein (SARS-CoV-nsp1ΔD-EΔ3) were generated. EΔ3 deletion mutant was selected for further studies because this deletion led to a virus that grew to titers similar or higher than the SARS-CoV-∆E, in cell culture or in mice, respectively (Fig 12A and 12B). More importantly, the E∆3 virus was genetically stable after 10 passages in cell culture [53] or *in vivo*, maintaining its attenuated phenotype (Fig 12C), in contrast to the ∆E virus (Figs 1 and 5). This deletion was combined with another one in SARS-CoV nsp1 protein (nsp1ΔD), which was fully attenuating. The resulting virus grew to relatively high titers *in vivo* (Figs 9 and 13A). Viruses were rescued in Vero E6 cells, cloned and sequenced to confirm the presence of the desired mutations.

To analyze the stability of the viruses in tissue culture cells, SARS-CoV-nsp1ΔD-ΔE and SARS-CoV-nsp1ΔD-EΔ3 were passaged 10 times in Vero E6 cells, followed by sequencing of the nsp1 and E genes. The deletions introduced in both nsp1 and E genes were conserved, suggesting that these deletions were genetically stable *in vitro*. SARS-CoV-nsp1ΔD-ΔE and SARS-CoV-nsp1ΔD-EΔ3 at passage 1 reached peak titers at 72 hpi (5·10^5^ pfu/ml and 5·10^4^ pfu/ml in Vero E6 and DBT-mACE2 cells, respectively) (Fig 13B). Decreased virus growth was likely due to deletions in the E gene, as previously described [53] (Fig 8B). After 10 passages, viruses showed a slight increase in titer (Fig 13B), suggesting incorporation of additional mutations but not in E or nsp1.

### Pathogenicity of a recombinant SARS-CoV including two safety guards in mice

**Fig 14:**
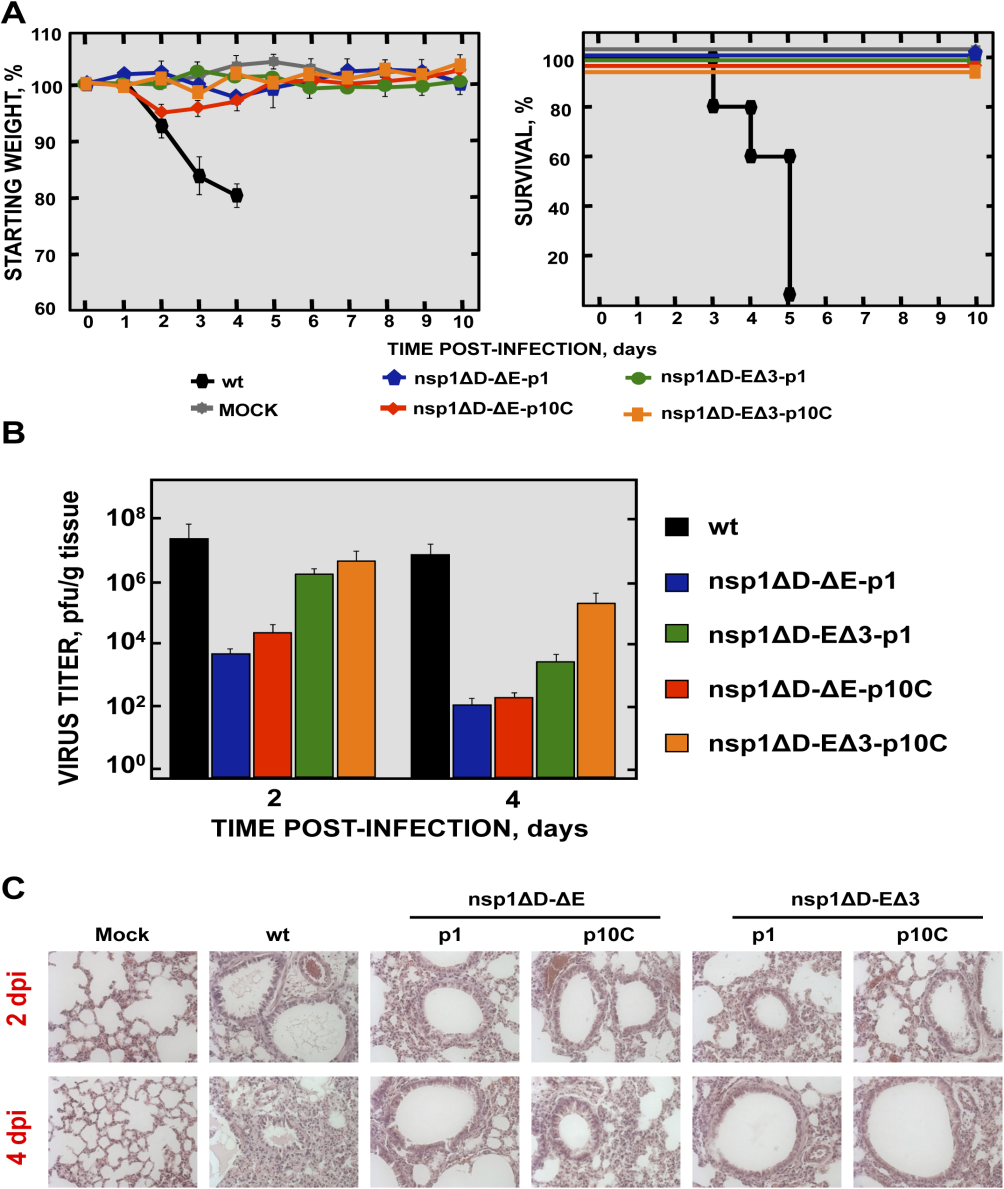
Virulence and virus growth of SARS-CoV including two safety guards. BALB/c mice were intranasally infected with 100,000 pfu of wt, SARS-CoV-nsp1ΔD-ΔE and SARS-CoV-nsp1ΔD-EΔ3 at passage 1 and 10 (-p1 and -p10C) viruses (5 mice per group). (A) Animals were monitored daily for weight loss and survival. (B) Viral titers in lungs were determined at 2 and 4 days post infection (3 mice per group and time). Error bars represent the standard deviations from three independent mice in each case. (C) Lung tissue sections from mice infected with the different recombinant viruses were prepared and stained with hematoxylin and eosin at 2 and 4 dpi. Three independent mice per group were analyzed. Original magnification was 20x and representative images are shown.

To analyze the pathogenicity of SARS-CoV-nsp1ΔD-ΔE and SARS-CoV-nsp1ΔD-EΔ3 mutants at passages 1 and 10 (p1 and p10C, respectively), BALB/c mice were intranasally inoculated with recombinant viruses. All mice infected with these viruses maintained their weight and survived (Fig 14A). In contrast, all mice infected with rSARS-CoV died (Fig 14A). These results indicated that viruses including deletions in both nsp1 and E proteins were attenuated *in vivo*. SARS-CoV-nsp1ΔD-EΔ3, especially the p10C virus, grew more efficiently than SARS-CoV-nsp1ΔD-ΔE (Fig 14B). Nevertheless, no obvious gross lesions or changes in weight were observed in the lungs of mice infected with any of these doubly mutant rSARS-CoV (Figs 14C and S4). In contrast, mice infected with rSARS-CoV showed lung injury and a marked increase in the weight of lungs, as described above (S4 Fig). Histological examination of lungs from SARS-CoV-nsp1ΔD-ΔE and SARS-CoV-nsp1ΔD-EΔ3 (p1 and p10C viruses)-infected mice showed only minimal evidence of damage or leukocyte infiltration at days 2 and 4 post-infection (Fig 14C) while rSARS-CoV-infected mice showed extensive cellular infiltration and edema. The rSARS-CoV-nsp1∆D-E∆3 was selected for further study because this virus showed higher titers *in vivo* as compared with the rSARS-CoV-nsp1∆D-∆E virus (Fig 14B), therefore it could promote a higher immunization. In addition, ∆E mutation led to unstable viruses that incorporated new chimeric proteins in cell culture, or a novel 8a protein *in vivo*, causing reversion to a virulent phenotype (Fig 6). In contrast, viruses containing the E∆3 mutation remained stable after 10 passages and maintained their attenuated phenotype (Fig 12).

### Stability and pathogenicity of doubly mutant rSARS-CoV during passage in mice

**Fig 15:**
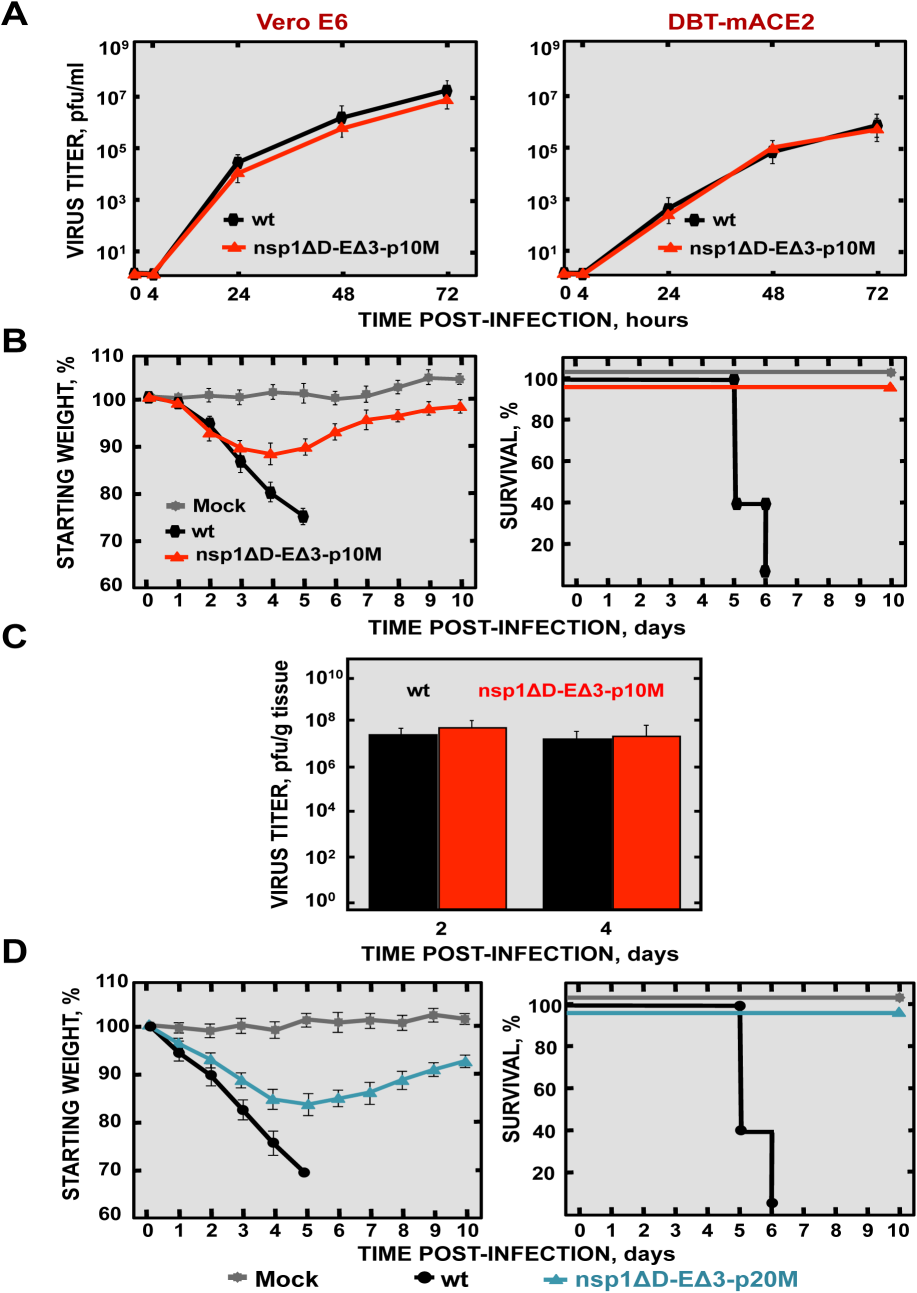
Virulence and virus growth of SARS-CoV with two safety guards after serial passage in mice. (A) Subconfluent monolayers of Vero E6 and DBT-mACE2 cells were infected with wt and SARS-CoV-nsp1ΔD-EΔ3-p10M at a moi of 0.001. At different times post infection, virus titers were determined by plaque assay on Vero E6 cells. Error bars represent standard deviations of the mean using data from three independent experiments. (B) BALB/c mice were intranasally infected with 100,000 pfu of wt virus, or SARS-CoV-nsp1ΔD-EΔ3-p1 and -p10M viruses (5 mice per group). Animals were monitored daily for weight loss and survival. (C) Viral titers in lungs were determined at 2 and 4 dpi (3 mice per group and time). Error bars represent the standard deviations from three independent mice in each case. (D) BALB/c mice were intranasally infected with 100,000 pfu of wt or SARS-CoV-nsp1ΔD-EΔ3-p20M viruses (5 mice per group). Animals were monitored daily for weight loss and survival.

To analyze the stability of rSARS-CoV with mutations in nsp1 and E in mice, SARS-CoV-nsp1ΔD-EΔ3 was passaged 10 times (p10M), followed by sequencing from the S gene to the 3´ end of the genome. Only two changes were observed in the viral sequence, one in the E gene (A26250T N→I), and a second one in the M gene (A26450G Q→R). The deletions introduced in the nsp1 and E genes were conserved, suggesting that this virus was essentially genetically stable *in vivo*. To analyze whether the E and M mutations could be compensatory mutations, virus titers at p1 and p10 were compared in Vero E6 and DBT-mACE2 cells. Titers of SARS-CoV-nsp1ΔD-EΔ3 (p10M) at different times post-infection were the same as those observed for rSARS-CoV (Fig 15A), indicating that the mutations increased virus replication.

To evaluate whether the compensatory mutations restored the pathogenicity of the virus, BALB/c mice were intranasally inoculated with rSARS-CoV and SARS-CoV-nsp1ΔD-EΔ3 (p10), and were daily monitored for 10 days. Mice infected with rSARS-CoV started to lose weight by day 2, and died by day 6 (Fig 15B). In contrast, although mice infected with the SARS-CoV-nsp1ΔD-EΔ3-p10M mutant initially lost 10% of their weight, at day 5 the mice started to regain weight, fully recovered from the disease, and 100% survived (Fig 15B). SARS-CoV-nsp1ΔD-EΔ3-p10M grew to similar titers as rSARS-CoV (Fig 15C). Nevertheless, unlike the parental virus, lungs of SARS-CoV-nsp1ΔD-EΔ3-p10M-infected mice presented no significant increase of inflammatory cytokines. In addition, no obvious lung lesions, weight increases, nor substantial inflammatory cell infiltration, as determined by macroscopic and histological examination, were observed (S5 Fig). To further support the stability and attenuation of the double mutant virus, additional passages (up to 20) were conducted in mice. Evaluation of the passaged virus virulence showed that rSARS-CoV-nsp1∆D-E∆3 remained attenuated (Fig 15D). These results indicated that despite the mutations that the virus incorporated after their passage in mice, the virus maintained the *in vivo* attenuated phenotype.

### Protection mediated by SARS-CoV-nsp1-ΔD-EΔ3

**Fig 16:**
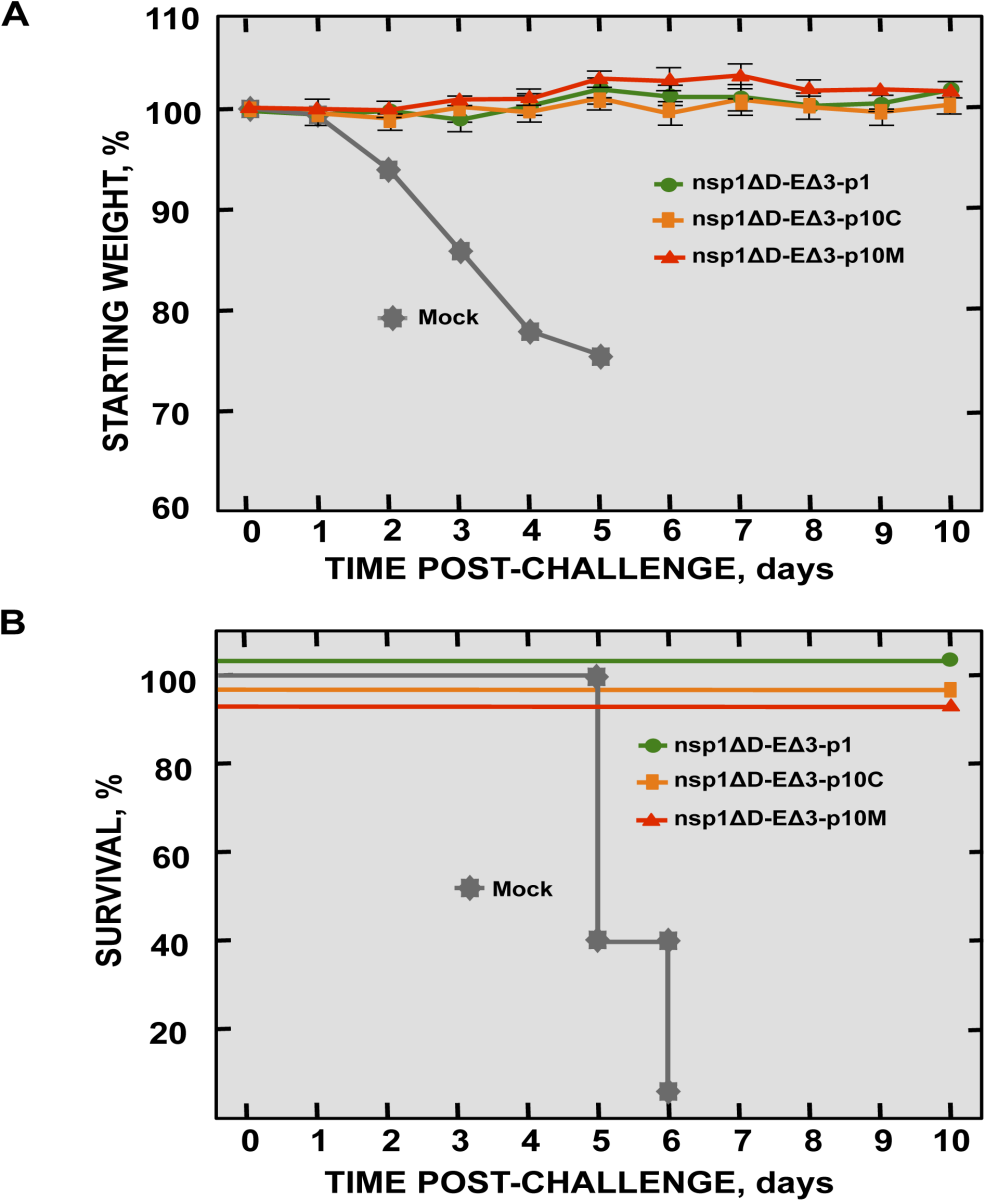
Protection conferred by immunization with SARS-CoV-double mutants. BALB/c mice (16-week-old) were mock-immunized or immunized with 6000 pfu of SARS-CoV-nsp1ΔD-EΔ3 viruses at passage 1 and 10 (-p1, -p10C and -p10M), and challenged at day 21 post-immunization with 100,000 pfu of wt virus (5 mice per group). Weight loss (A) and survival (B) were recorded daily.

To determine whether SARS-CoV-nsp1-ΔD-EΔ3 confers protection against challenge with rSARS-CoV, BALB/c mice were either immunized with SARS-CoV-nsp1ΔD-EΔ3-p1, -p10C and -p10M or non-immunized, as a control. At 21 days postimmunization, mice were challenged with rSARS-CoV administered by the same route. Non-immunized mice lost weight and all died on day 6 after the challenge (Fig 16A and 16B). In contrast, vaccination with the attenuated mutant viruses completely protected mice against challenge with rSARS-CoV (Fig 16A and 16B), indicating that the double mutant virus is a promising vaccine candidate.

## Discussion

We have previously shown that deletion of SARS-CoV E gene leads to an attenuated virus that is a promising vaccine candidate [30–34]. However, since safety and stability are main concerns of live attenuated vaccine candidates, we focused on rSARS-CoV-∆E stability *in vitro* and *in vivo* and on the generation of a safe vaccine candidate by identifying the mechanisms of reversion to virulence.

Unexpectedly, serial passage of rSARS-CoV-∆E in cell culture resulted in the generation of chimeric proteins composed of a partial duplication of the membrane gene fused to a part of the leader sequence. Our results are in agreement with a recombinant MHV lacking the E protein (rMHV-∆E) that was viable but its replication was drastically impaired [60]. rMHV-∆E replicated to 10,000-fold lower titers than the parental virus and remarkably, evolved similarly to SARS-CoV-∆E after serial passage in tissue culture cells [61]. Despite that the generation of a chimeric protein was previously observed in MHV when E gene was deleted [61], the presence of a PBM motif within the inserted chimeric sequence and its main role in providing genetic stability to SARS-CoV-ΔE during passage described in this manuscript, were not previously noticed. Alteration of coronavirus genome was not unexpected due to the high frequency of RNA recombination and mutation described for these viruses, both in cell culture and in animals [37, 62–64].

All chimeric SARS-CoV M proteins generated in cell culture were expressed, enhancing virus growth in cell culture compared to rSARS-CoV-∆E. Similarly, rMHV-∆E that contained chimeric proteins also showed significant increases in viral yields [61, 65]. In contrast, mice infected with rSARS-CoVs containing chimeric proteins generated in cell culture showed a decrease in viral titers in the lungs of infected mice even when compared with rSARS-CoV-∆E virus. Similarly, extensive passage in cell culture of other CoVs, including porcine epidemic diarrhea virus (PEDV) and transmissible gastroenteritis virus (TGEV), led to less pathogenic strains compared to wild-type viruses, possibly due to the emergence of deletion mutants that lost sequence domain not needed for their growth in cell culture, but that influenced their tropism and *in vivo* replication [66–68]. Chimeric proteins containing a PBM inserted in the viral genome after passage increased viral fitness in cell culture in a tissue specific manner, i.e., the chimeric protein inserted into viral genome during passage in monkey cells promoted virus growth in cells from this species, whereas the one inserted during passage in murine cells specifically increased virus fitness in cells from mice. Furthermore, these chimeric proteins did not enhance viral growth nor virulence *in vivo*. These data indicated that the insertion of chimeric proteins specifically adapted the virus for an optimum growth in cell culture but did not enhance *in vivo* growth nor virulence. In this context, it is also important to note that the activity of PBMs is dependent on their specific sequence, and also on the sequence context in which they are inserted [27, 28, 69, 70].

Serial passage of rSARS-CoV-∆E in mice introduced a partial duplication of 45 nucleotides in the 8a protein, resulting in its reversion to a virulent phenotype. This phenotype was associated to its ability to activate p38 MAPK and to the induction of inflammatory cytokine expression and increased lung damage, as previously described [24, 71].

8a protein is a short transmembrane protein composed of 39 amino acids that forms cation-selective ion channels [72]. SARS-CoV variants with deletions in 8a ORF, have been transmitted and maintained in humans in the late phases of SARS-CoV epidemic [73, 74]. Interestingly, an 8a protein mutant generated during virus passage *in vivo* contained a new potential PBM (CTTV) localized in the internal region of the carboxy-terminal domain of the protein (Fig 5). Despite the CTVV sequence was already present in the original 8a protein, it most likely does not represent a functional PBM, as it is located within the transmembrane domain of the 8a protein. Active PBMs are in general located in exposed regions of the proteins, usually the end of the carboxy-terminus or, exceptionally, in internal positions within the carboxy-terminal domain, allowing their interaction with PDZ domains, such as it has been observed in the NS5 proteins of tick-borne encephalitis virus (TBEV) and Dengue virus [75, 76]. However, PBMs forming part of a transmembrane domain are not accessible to PDZ-containing proteins and have not been described [29]. Therefore, the new CTVV sequence placed in an exposed environment may constitute a novel and active PBM. The PBM insertion within 8a protein after passaging *in vivo* could be due to the fact that the ORF8 is one of the regions where most variations were observed between human and animal isolates of SARS-CoV [4]. In fact, a complete genome sequence of SARS-like coronaviruses in bats isolates showed the presence of a PBM within the ORF8 [5]. As mentioned above, species of bats are a natural host of coronaviruses closely related to those responsible for the SARS outbreak.

PDZ domains are among the modules most frequently involved in protein-protein interactions found in all metazoans [69]. In the human genome, there are more than 900 PDZ domains in at least 400 different proteins [77]. Many pathogenic viruses produce PDZ ligands that disrupt host protein complexes for their own benefit, such as hepatitis B virus, influenza virus, rabies virus and human immunodeficiency virus, influencing their replication, dissemination in the host, transmission and virulence [29]. Generation of new proteins containing a PBM after passaging in cell culture and in mice may affect their interaction with a wide range of cellular PDZ-containing proteins, affecting diverse biological functions with high relevance in pathogenesis. E protein PBM participates in two different and independent issues, virus stability and virulence. Our data suggest that when the PBM was present in a proper environment at the end of the E protein, either a native or a mutant protein, viruses remained stable. An independent observation is that the presence of a PBM within E protein confers pathogenicity to the virus [24]. This virulence is prevented either by PBM removal [24] or by the introduction of small deletions within the carboxy-terminus of E protein [53], which by themselves may cause attenuation or, alternatively, by indirectly affecting the PBM. Our results highlighted the critical requirement of viral proteins containing a PBM in the generation of CoVs with virulent phenotypes, and opened up new approaches for the rational design of genetically stable vaccines.

Maintaining the attenuated phenotype of the vaccine candidate after passage *in vitro* was crucial to avoid the reversion to a virulent phenotype during the design and production of a genetically stable vaccine candidate. To this end, the identification of the relevance of the presence of a functional PBM motif at the carboxy-terminus of a transmembrane protein of the virus has been instrumental in the development of a stable SARS-CoV vaccine candidate. To minimize the risk of regain of virulence after passage, we engineered viruses with small deletions in E gene, instead of deletion of the entire E gene. By preserving the PBM, we observed no evidence for the development of chimeric proteins and thus no gain in virulence. As additional measures to ensure safety of this live attenuated vaccine candidate, we incorporated attenuating mutations into nsp1, in the context of the rSARS-CoV-ΔE or EΔ3. Nsp1 was chosen as a second attenuation target because this gene is located at a distant site (>20 kb) from that of the E gene in the viral genome, making it very unlikely that a single recombination event with a circulating wt coronavirus could result in the restoration of a virulent phenotype.

To analyze the role of SARS-CoV nsp1 in the pathogenesis of the virus, recombinant viruses encoding four different small deletions were generated. Deletion of amino acids 121–129 and 154–165, in the carboxy terminal region of nsp1 led to virus attenuation, indicating that nsp1 enhanced virus pathogenicity, as was previously shown for MHV [45, 48, 49]. Interestingly, these attenuated mutants grew in mice to lower titers than rSARS-CoV, probably by inducing higher IFN responses, indicating that these regions of nsp1 are critical for IFN antagonism. The induction of a higher innate immune response by the nsp1 deletion is most probably responsible for the decrease in SARS-CoV-nsp1* virus titers observed in mice and, to a lesser extent, in DBT-mACE–2 cells. In fact, a rSARS-CoV lacking the nsp1 protein grew poorly in IFN competent cells, but replicated as efficiently as the wt virus in IFN deficient cells [46], consistent with our findings. Similarly, titers of MHV deleted in nsp1 are restored almost to wild type levels in type I IFN receptor-deficient mice [48].

Immunization with singly deleted rSARS-CoV protected mice against challenge with rSARS-CoV, as it was previously shown with MHV nsp1-deletion mutants [48, 49]. SARS-CoV-nsp1ΔD-EΔ3, which contained deletions in nsp1 and E protein, maintained its attenuated phenotype after passage in Vero E6 cells and in mice. In addition, immunization with this double mutant fully protected mice from challenge with the parental virulent virus, indicating that it is a promising vaccine candidate in terms of both stability and efficacy.

Both humoral and cellular responses are relevant to protect from SARS [18, 19, 78, 79]. The viruses generated in this work express all viral proteins, except for small regions deleted in the E and nsp1 proteins, therefore have the potential of inducing both antibody and T cell responses, making this type of live vaccine more attractive than subunit or non replicating virus vaccines. Understanding of the molecular mechanisms by which an attenuated SARS-CoV reverted to a virulent phenotype could also be applied to the development of other relevant CoVs vaccines, such as MERS-CoV.

## Materials and Methods

### Ethics statement

Animal experimental protocols were approved by the Ethical Committee of The Center for Animal Health Research (CISA-INIA) (permit numbers: 2011–009 and 2011–09) in strict accordance with Spanish National Royal Decree (RD 1201/2005) and international EU guidelines 2010/63/UE about protection of animals used for experimentation and other scientific purposes and Spanish national law 32/2007 about animal welfare in their exploitation, transport and sacrifice and also in accordance with the Royal Decree (RD 1201/2005). Infected mice were housed in a ventilated rack (Allentown, NJ).

### Viruses

The mouse-adapted (MA15) [71] parental virus (wt), and recombinant viruses were rescued from infectious cDNA clones generated in a bacterial artificial chromosome (BAC) in our laboratory [32, 33, 53, 80].

### Cells

Vero E6 and BHK cells were kindly provided by E. Snijder (University of Leiden, The Netherlands) and H. Laude (Unité de Virologie et Immunologie Molecularies, INRA, France), respectively. The mouse delayed brain tumor (DBT) cells expressing the murine receptor (ACE2) for SARS-CoV (DBT-mACE2) were generated in our laboratory [38]. In all cases, cells were grown in Dulbecco's modified Eagle's medium (DMEM, GIBCO) supplemented with 25 mM HEPES, 2 mM L-glutamine (SIGMA), 1% non essential amino acids (SIGMA) and 10% fetal bovine serum (FBS, Biowhittaker). Virus titrations were performed in Vero E6 cells as previously described [33].

### Mice

8 week-old specific-pathogen-free BALB/c Ola Hsd mice females were purchased from Harlan Laboratories. BALB/c mice were maintained for 8 additional weeks in the animal care facility at the National Center of Biotechnology (Madrid). For infection experiments, mice were anesthetized with isoflurane and intranasally inoculated at the age of 16 weeks with 100,000 plaque forming units (pfu) of the indicated viruses. All work with infected animals was performed in a BSL3 laboratory (CISA, INIA).

### Generation of recombinant viruses

Mutant viruses (SARS-CoV-nsp1*) with small deletions covering different regions of nsp1 protein (SARS-CoV-nsp1-∆A, -∆B, -∆C and -∆D), were constructed using an infectious cDNA clone. cDNA encoding the genome of SARS-CoV-MA15 strain was assembled in a bacterial artificial chromosome (BAC) (plasmid pBAC-SARS-CoV-MA15) [32, 57, 80]. DNA fragments containing nucleotides 8142 to 9211, comprising the nsp1 gene of the SARS-CoV genome were generated by overlap extension PCR using as template the plasmid pBAC-SARS-CoV-MA15 and the primers indicated in S4 Table. The final PCR products were digested with the enzymes *AvrII* and *BstBI* and cloned into the intermediate plasmid pBAC-*SfoI*-*MluI*-SARS-CoV that contains the first 7452 nucleotides of the SARS-CoV infectious cDNA clone [80], to generate plasmids pBAC-*SfoI*-*MluI* SARS-CoV-nsp1* (pBAC-∆A, -∆B, -∆C, and -∆D) [80]. The plasmids pBAC-*SfoI*-*MluI*-SARS-CoV-nsp1* were digested with the restriction enzymes *SfoI* and *MluI* and the fragments were inserted into the plasmid pBAC-SARS-CoV-MA15, digested with the same restriction enzymes, to generate pBAC-SARS-CoV-MA15-nsp1* plasmids. Mutant viruses SARS-CoV-nsp1ΔD-ΔE and SARS-CoV-nsp1ΔD-EΔ3 were generated using the plasmids pBAC-SARS-CoV-MA15-ΔE and -EΔ3 [33, 53]. The plasmids were digested with the enzymes *BamHI* and *RsrII* and the digested fragments were exchanged with the fragment of plasmid pBAC-SARS-CoV-MA15-nsp1∆D, to generate pBAC-SARS-CoV-MA15-nsp1ΔD-ΔE and pBAC-SARS-CoV-MA15-nsp1ΔD-EΔ3 plasmids. Two fragments representing the nucleotides containing the chimeric proteins MCH-EPBM and 3aCH-3aPBM were chemically synthesized (BioBasic Inc) to generate SARS-CoV mutants. The final PCR products and synthesis fragments were digested with enzymes *BamHI* and *MfeI* and cloned into the intermediate plasmid psl1190+BamHI/SacII-SARS-CoV to generate the plasmids psl1190-∆E-MCH-EPBM and psl1190-∆E-3aCH-3aPBM. The plasmid psl1190+BamHI/SacII SARS-CoV contains a fragment corresponding to nucleotides 26045 to 30091 of the SARS-CoV infectious cDNA clone engineered into plasmid psl1190 (Pharmacia) [80]. These constructs were cloned in the infectious pBAC-SARS-CoV-MA15-∆E with the enzymes *BamHI* and *SacII*. Mutant virus rSARS-CoV-∆E-8a-dup with small duplication of 8a protein was constructed using an infectious cDNA clone. cDNA encoding the genome of SARS-CoV-MA15-∆E strain was assembled in a bacterial artificial chromosome (BAC) (plasmid pBAC-SARS-CoV-MA15-∆E) [32, 57, 80]. DNA fragments containing nucleotides 27779 to 27898, comprising the 8a gene of the SARS-CoV genome were generated by overlap extension PCR using as template the plasmid pBAC-SARS-CoV-MA15 and the primers indicated in S4 Table. The final PCR products were digested with the enzymes *XcmI* and *NheI* and cloned into the intermediate plasmid pBAC-*BamHI*-*NheI*-SARS-CoV that contains the nucleotides (nt) 26044 to 28753 nucleotides of the SARS-CoV infectious cDNA clone [80], to generate plasmid pBAC-*BamHI*-*NheI*-SARS-CoV-∆E-8a-dup [80]. The plasmid pBAC-*BamHI*-*NheI*-SARS-CoV-∆E-8a-dup was digested with the restriction enzymes *BamHI* and *NheI* and the fragments were inserted into the plasmid pBAC-SARS-CoV-MA15, digested with the same restriction enzymes, to generate pBAC-SARS-CoV-MA15-∆E-8a-dup plasmid. The viruses were rescued in BHK and Vero E6 cells as previously described [33]. Viruses were cloned by three rounds of plaque purification.

### Growth kinetics

Subconfluent monolayers (90% confluency) of Vero E6 and DBT-mACE2 on 12.5 cm^2^ flasks were infected at a multiplicity of infection (moi) of 0.001 with the indicated viruses. Culture supernatants were collected at 0, 4, 24, 48 and 72 hpi and virus titers were determined as previously described [33].

### Virus infection and growth in mice

BALB/c mice were anesthetized with isoflurane and intranasally inoculated with 100,000 plaque forming units (pfu) of virus in 50 μL of DMEM. Weight loss and mortality were evaluated daily. For protection experiments mice were immunized intranasally with 6000 pfu of the attenuated viruses, and then challenged with an intranasal inoculation of 100,000 pfu of SARS-CoV at 21 days post-immunization. Mice were monitored daily for weight loss and mortality. To determine SARS-CoV titers, lungs were homogenized in PBS containing 100 UI/ml penicillin, 0.1 mg/ml streptomycin, 50 μg/ml gentamicin, and 0.5 μg/ml amphotericin B (Fungizone), using a *gentleMACS* dissociator (Miltenyi Biotec) and virus titrations were performed in Vero E6 cells as described above. Viral titers were expressed as pfu/g tissue.

### Histopathology

Mice were sacrificed at 2 and 4 dpi. Lungs were removed, fixed in 10% zinc formalin for 24 h at 4°C and paraffin embedded. Histological examination was performed using hematoxylin and eosin staining of sections.

### Serial passage of SARS-CoV in mice

BALB/c mice were anesthetized with isoflurane and intranasally inoculated with 100,000 pfu of the indicated recombinant viruses in 50 μL of DMEM. Two days after inoculation, mice were euthanized, and their lungs were removed and homogenized as previously described. The lung homogenate was clarified by low-speed centrifugation at 3,000 rpm for 12 min, and 100 μL of the supernatant was administered intranasally to naive mice. Intranasal inoculation of BALB/c mice with clarified supernatants of lung homogenates collected 2 dpi was repeated 10 times.

### Western blot analysis

Cell lysates were resolved by sodium dodecyl sulfate-polyacrylamide gel electrophoresis (SDS-PAGE), transferred to a nitrocellulose membrane by wet immunotransfer and processed for Western blotting. The blots were probed with monoclonal antibodies for p38 MAPK (dilution 1:500; Cell Signaling), phospho-p38 MAPK (dilution 1:500; Cell Signaling) and actin (dilution 1:10,000; Abcam) or polyclonal antibodies specific for M (dilution 1:1000; Biogenes) and MCH-DBT (dilution 1:1000; Biogenes) proteins. Both polyclonal antibodies recognizing the parental SARS-CoV M protein or the MCH-DBT protein were generated by Biogenes (Germany) as previously described [81] using synthetic peptides corresponding to the residues RTRSMWSFNPETNILLNVPLRGTIVTRPLM and PLMNLSLVL, respectively. Bound antibodies were detected with horseradish peroxidase-conjugated goat anti-rabbit (dilution 1:30,000; Cappel) and the Immobilon Western chemiluminescent substrate (Millipore).

### RNA analysis by RT-qPCR

DBT-mACE2 cells were infected with SARS-CoV, SARS-CoV-nsp1-ΔC and -ΔD at a moi of 0.125. Total RNAs from DBT-mACE2 infected cells were extracted at 48 hpi using the Qiagen RNeasy kit according to the manufacturer’s instructions. Quantitative reverse transcription-polymerase chain reaction (qRT-PCR) reactions were performed at 37°C for 2 h using the High Capacity cDNA transcription kit (Applied Biosystems) and 100 ng of total RNA and random hexamer oligonucleotides. Cellular gene expression was analyzed using TaqMan gene expression assays (Applied Biosystems) specific for *Mus musculus* genes (S3 Table). Data were acquired with an ABI PRISM 7000 sequence detection system (Applied Biosystems) and analyzed with ABI PRISM 7000 SDS version 1.0 software. Gene expression in mock-infected cells and SARS-CoV, SARS-CoV-nsp1-ΔC and -ΔD-infected cells was compared. Quantification was achieved using the 2^-ΔΔCt^ method, which analyzes relative changes in gene expression in qPCR experiments (Livak and Scmittgen, 2001). The results of three independent experiments were analyzed. All experiments and data analysis were MIQE compliant [82].

### Cytokine expression analysis from lung samples using RT-qPCR

Lung sections from infected animals were collected at 2 dpi and homogenized using gentleMACS Dissociator (Miltenyibiotec). Then, total RNA was extracted using the RNeasy purification kit (Qiagen). Reactions were performed at 37°C for 2 h using a High Capacity cDNA transcription kit (Applied Biosystems) with 100 ng of total RNA and random hexamer oligonucleotides. Cellular gene expression was analyzed using TaqMan gene expression assays (Applied Biosystems) specific for mouse genes (S4 Table). Data representing the average of three independent experiments were acquired and analyzed as previously described [57]. All experiments and data analysis were MIQE compliant [82].

### Computational structure predictions

The computer modeling of 8a protein structure was performed with the raptorX server http://raptorx.uchicago.edu [83]. The predicted structures were visualized using Pymol (http://www.pymol.org/).

### Statistical analysis

Student´s *t* test was used to analyze differences in mean values between groups. All results are expressed as means ± standard errors of the means. *P* values of <0.05 were considered statistically significant.

### Accession numbers

The UniProt (http://www.uniprot.org/) accession numbers for genes and proteins discussed in this paper are: SARS-CoV E protein, P59637; SARS-CoV 8a protein, Q19QW2; SARS 3a protein, P59632; SARS M protein, P59596; mouse IFN-β, P01575; mouse IRF1, P15314; mouse DDX58, Q6Q899; mouse STAT1, P42225; human p38 MAPK, Q16539; human ACE2, Q9BYF1; mouse ACE2, Q8R0I0; mouse CXCL10, P17515; mouse CCL2, P10148; mouse IL6, P08505; mouse 18S, O35130; human actin, P60709.

## Supporting Information

S1 Fig Synthesis of sgmRNAs corresponding to chimeric genes generated after SARS-CoV-∆E passage in cell culture. Vero E6 cells were mock-infected or infected with the parental (wt) and SARS-CoV-∆E (∆E) viruses at passage 1 (p1) or 16 (p16) at a moi of 0.3. Expression of sgmRNAs was analyzed at 24 hpi using specific primers. (A) Representation of chimeric genes sgmRNAs generated after ∆E passage in Vero E6 (MCH-Vero) and DBT-mACE2 (MCH-DBT) cells. Position of specific forward (VS) and reverse (RS) primers is shown with arrows. Leader sequence is represented in green, TRSs are shown in light red and the sequence corresponding to chimeric genes is shown in red and blue boxes. Dark and light blue boxes indicate the different specific sequences. (B) PCR products in mock-cells or cells infected with the different viruses at passage 1 and 16 using specific primers (S2 Table). Vero E6 cells were mock-infected or infected with the wt, ∆E, MCH-Vero and MCH-DBT viruses at an initial moi of 0.3.
(TIF)

S2 Fig Lung weight and pathology of mice infected with recombinant SARS-CoV-∆E viruses after serial passage in cell culture. 16-week-old BALB/c mice were intranasally inoculated with 100,000 pfu of wt, ΔE, MCH-Vero and MCH-DBT viruses. (A) Gross pathology of mouse lungs infected with recombinant viruses at 2 and 4 dpi. (B) Weight of left lungs excised from infected mice, sacrificed at the indicated days (n = 3, each day). Error bars represent standard deviations. Statistically significant data are indicated with two asterisks (*P* < 0.01).
(TIF)

S3 Fig Lung weight and pathology of mice infected with recombinant SARS-CoVs containing deletions within nsp1 protein. 16-week-old BALB/c mice were intranasally inoculated with 100,000 pfu of wt, ΔA, ΔB, ΔC and ΔD viruses. (A) Gross pathology of mouse lungs infected with recombinant viruses at 2 and 4 dpi. (B) Weight of left lungs excised from infected mice, sacrificed at the indicated days (n = 3, each day). Error bars represent standard deviations. Statistically significant data are indicated with two asterisks (*P* < 0.01).
(TIF)

S4 Fig Lung pathology caused by infection with SARS-CoV including two safety guards. 16-week-old BALB/c mice were intranasally inoculated with 100,000 pfu of wt and the indicated SARS-CoV double mutants. (A) Gross pathology of mouse lungs infected with recombinant viruses at 2 and 4 dpi. (B) Weight of left lungs excised from infected mice, sacrificed at the indicated days (n = 3, each day). Error bars represent standard deviations. Statistically significant data are indicated with two asterisks (*P* < 0.01).
(TIF)

S5 Fig Lung weight, pathology and inflammatory cytokines expression during infection with recombinant nsp1ΔD-EΔ3 virus after serial passage in mice. 16-week-old BALB/c mice were intranasally inoculated with 100,000 pfu of wt or nsp1ΔD-EΔ3-p10M viruses. (A) Gross pathology of mouse lungs infected with recombinant viruses at 2 and 4 dpi. (B) Weight of left lungs excised from infected mice, sacrificed at the indicated days (n = 3, each day). Error bars represent standard deviations. Statistically significant data are indicated with two asterisks (*P* < 0.01). (C) Lung tissue sections from mice infected with the different recombinant viruses were prepared and stained with hematoxylin and eosin at 2 and 4 dpi. Three independent mice per group were analyzed. Original magnification was 20x and representative images are shown. (D) Expression of inflammatory cytokines in lungs of infected mice evaluated by RT-qPCR at 2 dpi. Three independent experiments were analyzed with similar results in all cases. Error bars represent the means of three experiments analyzed for each condition. Statistically significant data compared to nsp1ΔD-EΔ3-p10M-infected cells are indicated with one (*P* < 0.05) asterisk.
(TIF)

S1 Table Specific primers used to sequence the distal third of the SARS-CoV genome. (DOCX)

S2 Table Specific primers used to sequence sgmRNAs corresponding to chimeric genes. (DOCX)

S3 Table Taqman assays used to analyze the expression of cellular genes by quantitative RT-PCR. (DOCX)

S4 Table Primers used for the generation of SARS-CoV-nsp1* protein deletion mutants and SARS-CoV-∆E-8a-dup mutant. (DOCX)
