# Supplementary figures and images for: Identification of the Mechanisms Causing Reversion to Virulence in an Attenuated SARS-CoV for the Design of a Genetically Stable Vaccine
Source: PLoS Pathog. 2015 Oct 29;11(10):e1005215. doi: 10.1371/journal.ppat.1005215 (PMC4626112; doi:10.1371/journal.ppat.1005215)

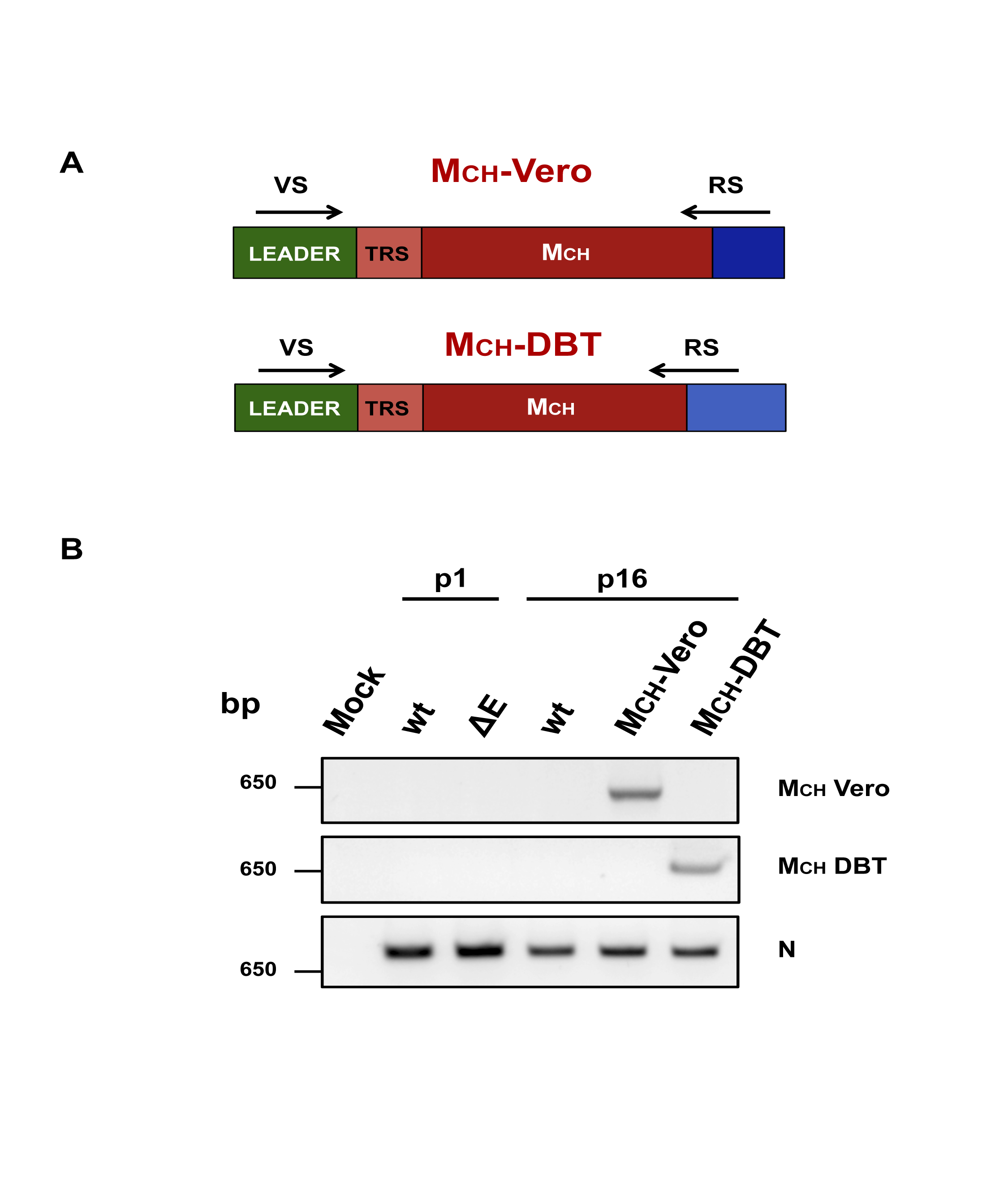

Supplement: S1 Fig — Vero E6 cells were mock-infected or infected with the parental (wt) and SARS-CoV-∆E (∆E) viruses at passage 1 (p1) or 16 (p16) at a moi of 0.3. Expression of sgmRNAs was analyzed at 24 hpi using specific primers. (A) Representation of chimeric genes sgmRNAs generated after ∆E passage in Vero E6 (MCH-Vero) and DBT-mACE2 (MCH-DBT) cells. Position of specific forward (VS) and reverse (RS) primers is shown with arrows. Leader sequence is represented in green, TRSs are shown in light red and the sequence corresponding to chimeric genes is shown in red and blue boxes. Dark and light blue boxes indicate the different specific sequences. (B) PCR products in mock-cells or cells infected with the different viruses at passage 1 and 16 using specific primers (S2 Table). Vero E6 cells were mock-infected or infected with the wt, ∆E, MCH-Vero and MCH-DBT viruses at an initial moi of 0.3. (TIF) [file ppat.1005215.s001.tif]

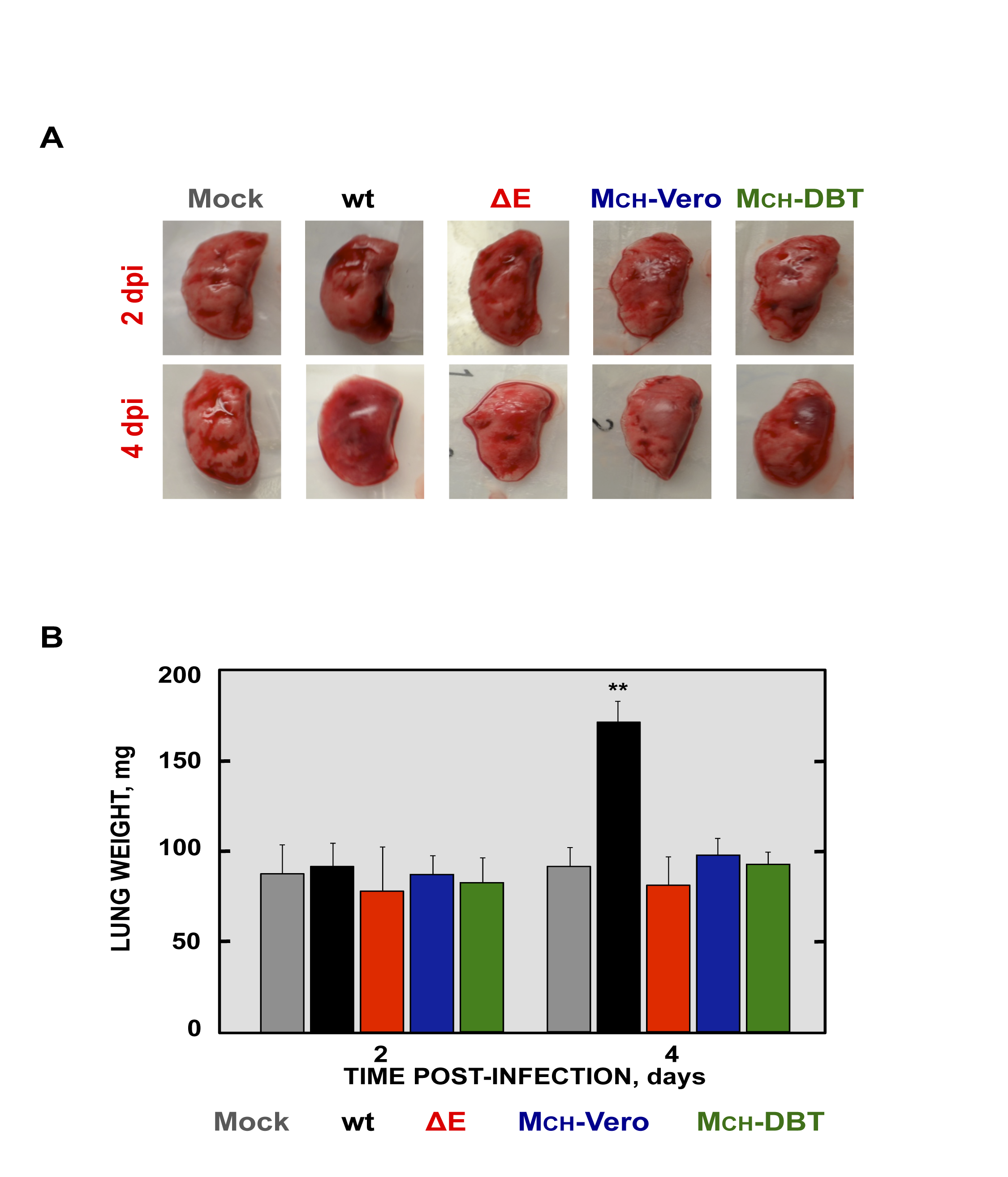

Supplement: S2 Fig — 16-week-old BALB/c mice were intranasally inoculated with 100,000 pfu of wt, ΔE, MCH-Vero and MCH-DBT viruses. (A) Gross pathology of mouse lungs infected with recombinant viruses at 2 and 4 dpi. (B) Weight of left lungs excised from infected mice, sacrificed at the indicated days (n = 3, each day). Error bars represent standard deviations. Statistically significant data are indicated with two asterisks (P < 0.01). (TIF) [file ppat.1005215.s002.tif]

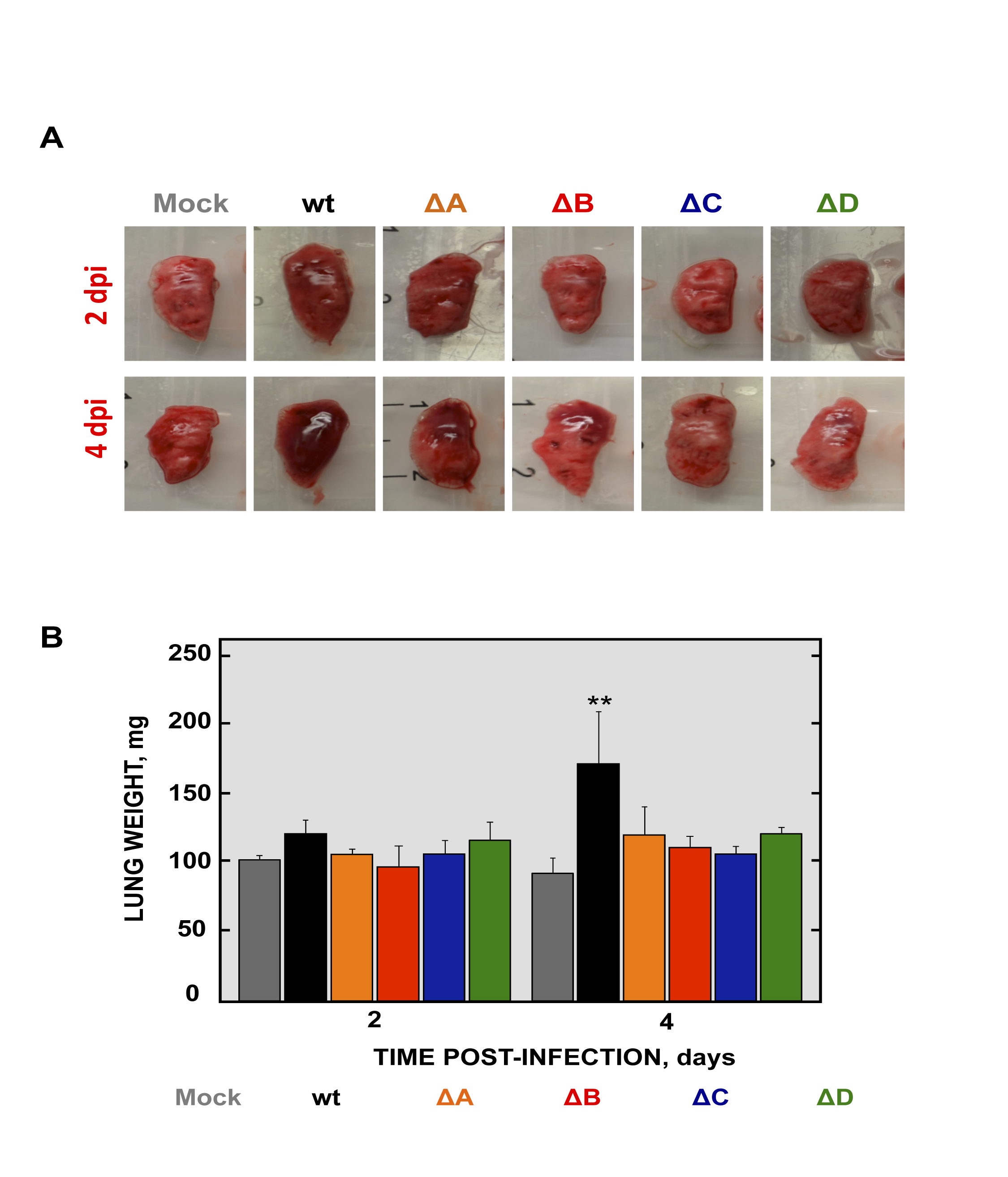

Supplement: S3 Fig — 16-week-old BALB/c mice were intranasally inoculated with 100,000 pfu of wt, ΔA, ΔB, ΔC and ΔD viruses. (A) Gross pathology of mouse lungs infected with recombinant viruses at 2 and 4 dpi. (B) Weight of left lungs excised from infected mice, sacrificed at the indicated days (n = 3, each day). Error bars represent standard deviations. Statistically significant data are indicated with two asterisks (P < 0.01). (TIF) [file ppat.1005215.s003.tif]

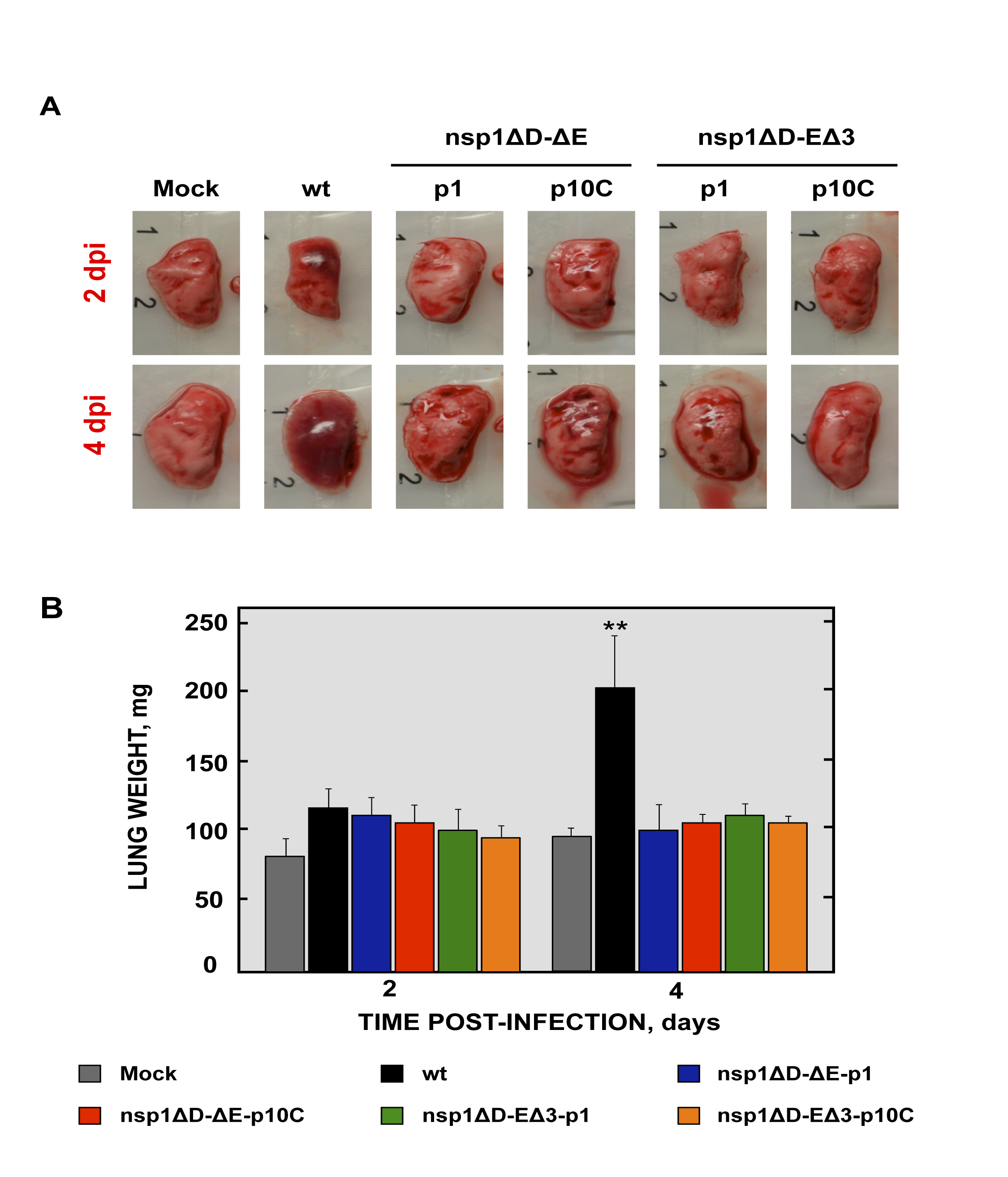

Supplement: S4 Fig — 16-week-old BALB/c mice were intranasally inoculated with 100,000 pfu of wt and the indicated SARS-CoV double mutants. (A) Gross pathology of mouse lungs infected with recombinant viruses at 2 and 4 dpi. (B) Weight of left lungs excised from infected mice, sacrificed at the indicated days (n = 3, each day). Error bars represent standard deviations. Statistically significant data are indicated with two asterisks (P < 0.01). (TIF) [file ppat.1005215.s004.tif]

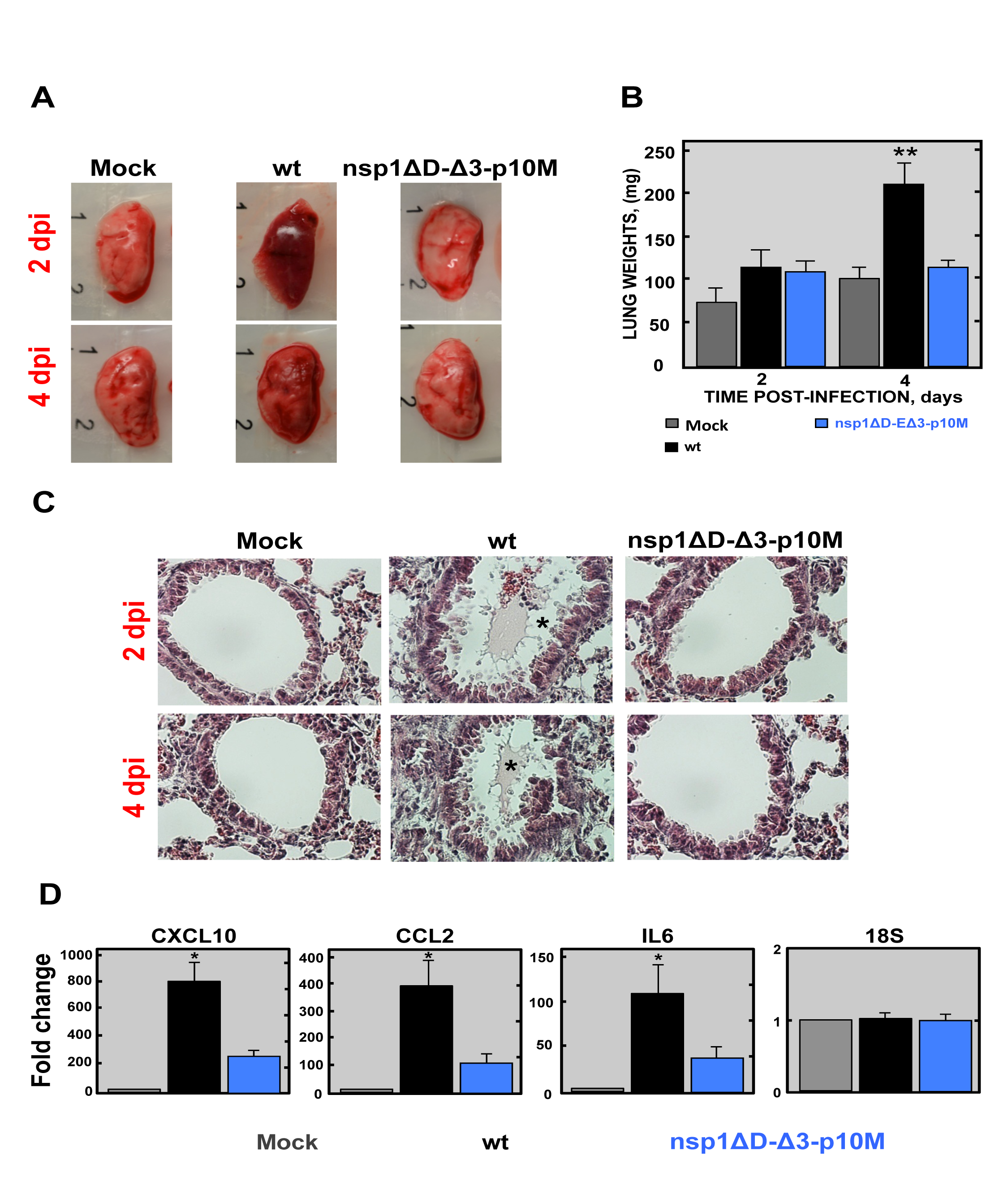

Supplement: S5 Fig — 16-week-old BALB/c mice were intranasally inoculated with 100,000 pfu of wt or nsp1ΔD-EΔ3-p10M viruses. (A) Gross pathology of mouse lungs infected with recombinant viruses at 2 and 4 dpi. (B) Weight of left lungs excised from infected mice, sacrificed at the indicated days (n = 3, each day). Error bars represent standard deviations. Statistically significant data are indicated with two asterisks (P < 0.01). (C) Lung tissue sections from mice infected with the different recombinant viruses were prepared and stained with hematoxylin and eosin at 2 and 4 dpi. Three independent mice per group were analyzed. Original magnification was 20x and representative images are shown. (D) Expression of inflammatory cytokines in lungs of infected mice evaluated by RT-qPCR at 2 dpi. Three independent experiments were analyzed with similar results in all cases. Error bars represent the means of three experiments analyzed for each condition. Statistically significant data compared to nsp1ΔD-EΔ3-p10M-infected cells are indicated with one (P < 0.05) asterisk. (TIF) [file ppat.1005215.s005.tif]
