# Supplementary material for: Identification of the Mechanisms Causing Reversion to Virulence in an Attenuated SARS-CoV for the Design of a Genetically Stable Vaccine
Source: PLoS Pathog. 2015 Oct 29;11(10):e1005215. doi: 10.1371/journal.ppat.1005215 (PMC4626112; doi:10.1371/journal.ppat.1005215)
Supplement: S1 Table — (DOCX) [file ppat.1005215.s006.docx]

**S1 Table. Specific primers used to sequence the distal third of the SARS-CoV genome.**

| **PCR** | **Primer** | **Sequence** | **Lenght (nt)** | **Product size (pb)** |
| --- | --- | --- | --- | --- |
| 1 | S1-VS  S1210-RS | ATGTTTATTTTCTTATTATTTC  CACCAGTTTGTCCTGGCGC | 22  19 | 1210 |
| 2 | S1002-VS  S2243-RS | CCCTTCTGTCTATGCATGGG  CGACGATTTAGTTGTGTGC | 20  19 | 1242 |
| 3 | S1943-VS  S2797-RS | GCGACATTCCTATTGGAGCTGGC  CGTCTTGCAGCTTGCCCAATGC | 23  22 | 855 |
| 4 | S2500-VS  S3768-RS | GCGCAGAAGTTCAATGGAC  TTATGTGTAATGTAATTTGACACCC | 19  25 | 1269 |
| 5 | S3581-VS  E231-RS | GGCCTTGGTATGTTTGGCTCGGC  TTAGACCAGAAGATCAGGAACTCC | 23  24 | 1275 |
| 6 | E-VS  27244-RS | CTCTTCAGGAGTTGCTAATCCAGCAATGGGGTTCTTCATCATGTAACTCCG | 29  22 | 1228 |
| 7 | 27024-VS  28397-RS | CGCCGGTAGCAACGACAATATTGC  GGGTAGCTCTTCGGTAGTAGCC | 24  22 | 1374 |
| 8 | N1-VS  N1269-RS | ATGTCTGATAATGGACCCC  TTATGCCTGAGTTGAATCTGC | 19  21 | 1269 |
| 9 | N611-VS  3’-end-RS | GGGGAAATTCTCCTGCTCG  GTCATTCTCCTAAGAAGCTATTAAAATCAC | 19  30 | 875 |
