# Supplementary material for: Identification of the Mechanisms Causing Reversion to Virulence in an Attenuated SARS-CoV for the Design of a Genetically Stable Vaccine
Source: PLoS Pathog. 2015 Oct 29;11(10):e1005215. doi: 10.1371/journal.ppat.1005215 (PMC4626112; doi:10.1371/journal.ppat.1005215)
Supplement: S2 Table — (DOCX) [file ppat.1005215.s007.docx]

**S2 Table. Specific primers used to sequence sgmRNAs corresponding to chimeric genes.**

| **PCR** | | **Primer** | **Sequence** | **Lenght (nt)** | **Product size (pb)** |
| --- | --- | --- | --- | --- | --- |
| MCH-Vero | | Urb-29-VS  MQ1-RS | AAGCCAACCAACCTCGATCTC AGAGAACAGATCTACAAGGT | 21  19 | 564 |
| MCH-DBT | Urb-29-VS  MQ2-RS | | AAGCCAACCAACCTCGATCTC TGGGTAGGTAAAAACGTGAT | 21  20 | 555 |
| N | Urb-29-VS  N733-RS | | AAGCCAACCAACCTCGATCTC GGCCTTGTTGTTGTTGGCC | 21  19 | 782 |
