# Supplementary material for: Identification of the Mechanisms Causing Reversion to Virulence in an Attenuated SARS-CoV for the Design of a Genetically Stable Vaccine
Source: PLoS Pathog. 2015 Oct 29;11(10):e1005215. doi: 10.1371/journal.ppat.1005215 (PMC4626112; doi:10.1371/journal.ppat.1005215)
Supplement: S3 Table — (DOCX) [file ppat.1005215.s008.docx]

**S3 Table. Taqman assays used to analyze the expression of cellular genes by quantitative RT-PCR.**

| **Gene name** | **Taqman assay*** | **Description** |
| --- | --- | --- |
| *CXCL10/IP-10* | Mm00445235-m1 | Interferon inducible protein 10 |
| *CCL2/MCP-1* | Mm00441242-m1 | Monocyte chemotactic protein 1 |
| *IL-6* | Mm00446190-m1 | Interleukin 6 |
| IFN-β | Mm00439552-s1 | Interferon β |
| IRF1 | Mm01288580_m1 | Interferon regulatory factor 1 |
| DDX58 | Mm01216853_m1 | DEAD (Asp-Glu-Ala-Asp) box polypeptide |
| STAT1 | Mm00439531_m | Signal transducer and activator of transcription 1 |
| *18S* | Mm03928990-g1 | 18S ribosomal RNA |

*Mm, *Mus musculus*.
