# Supplementary material for: Identification of the Mechanisms Causing Reversion to Virulence in an Attenuated SARS-CoV for the Design of a Genetically Stable Vaccine
Source: PLoS Pathog. 2015 Oct 29;11(10):e1005215. doi: 10.1371/journal.ppat.1005215 (PMC4626112; doi:10.1371/journal.ppat.1005215)
Supplement: S4 Table — (DOCX) [file ppat.1005215.s009.docx]

**S4 Table. Primers used for the generation of SARS-CoV-nsp1* protein deletion mutants** **and** **SARS-CoV-∆E-8a-dup mutant.**

| **mutant*** | **PCR** | **Primer** | **Sequence** |
| --- | --- | --- | --- |
| ΔA  24 nt | 1 | SARS-8142-VS  SARS-9211-RS | GCAGTCGATCATCAGCATACCTAGGTTTCGTCCGGGTGTGACCGAAAGGTAAGATGGAGAGCCTTGTTCTTCAACTCAGTTTGCCTGTCCTTCAGG  GGCTTTCAGAAAGTCGCACGTCTGCC |
| ΔB  33 nt | 1  2 | SARS-8028-VS  E SARS-8471-RS  SARS-8450-VS  SARS-9211-RS | GGCTGCATGCCTAGTGCACCTACGC  AGAACGTTTAATGAACACATAGGGCCCATTTCTGCAACCAGCTCAAC  GTTGAGCTGGTTGCAGAAATGG  GGCTTTCAGAAAGTCGCACGTCTGCC |
| ΔC  27 nt | 1  2 | SARS-8028-VS  SARS-8609-RS  SARS-8585-VS  SARS-9211-RS | GGCTGCATGCCTAGTGCACCTACGC  AACATTGCGGTATGCAATTGGGGCGATGCCATAGCTATGACCACCGGC  GCCGGTGGTCATAGCTATGGCATCG  GGCTTTCAGAAAGTCGCACGTCTGCC |
| ΔD  33 nt | 1  2 | SARS-8028-VS  SARS-8713-RS  SARS-8690-VS  SARS-9211-RS | GGCTGCATGCCTAGTGCACCTACGC  AATGGGATCAGTGCCAAGCTCGTCACCGAGTTCACGGAGTGCACCACTGCC  GGCAGTGGTGCACTCCGTGAACTC  GGCTTTCAGAAAGTCGCACGTCTGCC |
| ∆E-8a-dup  45 nt | 1  2 | SARS-26766-VS  SARS-2516-RS  SARS-2452-VS  SARS-27753-RS | CCGGGGGACAATTGTGACCAGAC  AGCACATGAGGTTTATTAGATGCACAGCGCTGTACTACAGTGCATATGCAACTGCATAGAGAAATACAAGTCAAAACAATGAG  CTCATTGTTTTGACTTGTATTTCTCTATGCAGTTGCATATGCACTGTAGTACAGCGCTGTGCATCTAATAAACCTCATGTGCT  GGGCAGTTTCACCACCTCCGCTAGC |
